# *Pfaffia glomerata* Ameliorates BPA-Induced Reproductive Impairments in Mice by Suppressing Apoptosis via PI3K/AKT Signaling Activation

**DOI:** 10.3390/ph18111614

**Published:** 2025-10-25

**Authors:** Hongwei Xue, Shuyan Zhang, Juan Lu, Jia Liu, Yihang Li, Xi Chen

**Affiliations:** 1 State Key Laboratory for Quality Ensurance and Sustainable Use of Dao-di Herbs, Institute of Medicinal Plant Development, Chinese Academy of Medical Sciences, Peking Union Medical College, Beijing 100193, China; 15297318065@163.com (H.X.); 15895057558@163.com (S.Z.); jlu@implad.ac.cn (J.L.); lj200929@126.com (J.L.); 2 Yunnan Branch, Institute of Medicinal Plant Development, Chinese Academy of Medical Sciences, Jinghong 666100, China

**Keywords:** *Pfaffia glomerata*, network pharmacology, reproductive system injury, cell apoptosis, PI3K/AKT signaling pathway

## Abstract

**Objectives:** Bisphenol A (BPA), a prototypical environmental endocrine-disrupting chemical (EDC), is ubiquitously present in environmental matrices and biological fluids. Dietary ingestion and inhalation exposure to BPA can induce testicular oxidative stress and apoptosis. This study aimed to investigate the protective effects and underlying mechanisms of *Pfaffia glomerata* (Pg), a perennial herb of the Amaranthaceae family, against BPA-induced reproductive system injury. **Methods:** Potential targets and molecular mechanisms were predicted through network pharmacology. Physiological indicators, histopathological changes, serum biochemical parameters, and Western blot analysis were used to systematically evaluate the ameliorative effects of Pg and elucidate its mechanisms. **Results:** Our network pharmacology analysis identified core targets of Pg in attenuating reproductive system injury, including PTPN11, PIK3CA, JAK2, PIK3R1, PDGFRB, and others. GO enrichment and KEGG pathway analysis indicated that these key targets primarily regulate steroid metabolism, enhance antioxidant capacity, and modulate signaling pathways such as PI3K-AKT, Fc epsilon RI, and cAMP. In vivo studies demonstrated that all Pg dose groups showed significant improvement in BPA-induced histopathological injury to testicular tissues. BPA exposure increased serum levels of follicle-stimulating hormone (FSH) while decreasing testosterone (T), estradiol (E2), and progesterone (PROG) levels. Furthermore, BPA elevated serum levels of the testicular marker enzymes acid phosphatase (ACP) and lactate dehydrogenase (LDH) but reduced alkaline phosphatase (ALP) levels; all these effects were significantly reversed with Pg treatment. Western blot results showed that compared with the model group, high-dose Pg significantly upregulated the expression of phosphorylated AKT (p-AKT), phosphorylated PI3K (p-PI3K), and Bcl-2, while downregulating Cleaved Caspase-3 and Bax. **Conclusions:** Our findings indicate that Pg may attenuate BPA-induced reproductive system injury by activating the PI3K/AKT signaling pathway, upregulating the anti-apoptotic protein Bcl-2, and inhibiting the activation of the apoptotic effector Caspase-3. The study provides a new theoretical basis for the development of novel natural drugs or health products.

## 1. Introduction

EDCs are environmentally persistent exogenous compounds that can disrupt endocrine homeostasis by mimicking, antagonizing, or interfering with physiological processes such as hormone synthesis, secretion, transport, and signaling pathways [1]. Since the U.S. Environmental Protection Agency (EPA) first formally defined “endocrine disruptors” in 1996, subsequent revisions by the Endocrine Society have established the current consensus definition: “exogenous chemicals or mixtures that interfere with any aspect of hormone action, including synthesis, release, transport, metabolism, binding, or elimination” [2]. As persistent organic pollutants (POPs), EDCs are pervasive in consumer products, including plastics (e.g., BPA), cosmetics (e.g., phthalates), industrial additives (e.g., PFASs), and pesticides (e.g., organochlorines), while exhibiting hormone-mimicking properties and bioaccumulative potential. Among diverse EDCs, bisphenols, phthalates, polychlorinated biphenyls (PCBs), and organophosphorus flame retardants pose significant health risks due to their environmental persistence and bioaccumulative potential [3,4,5]. These compounds enter the human body through dietary intake, inhalation, and dermal absorption, accumulating in adipose tissue as lipophilic reservoirs that prolong their half-lives and facilitate chronic exposure. Notably, BPA, one of the highest-volume EDCs, is primarily used in polycarbonate plastics and epoxy resins (e.g., food/beverage containers, electronic housings). Its potent endocrine-disrupting effects and multi-pathway exposure mechanisms pose substantial threats to human health [6,7,8]. Meanwhile, evidence for the endocrine-disrupting effects of BPA continues to grow, prompting stricter regulations in recent years. As a result, the industry has increasingly adopted substitutes such as bisphenol S (BPS), bisphenol F (BPF), and bisphenol AF (BPAF). These structurally similar analogs, however, not only exhibit comparable toxicity but may also present similar or even greater pharmacokinetic risks [9,10]. For example, some studies indicate that BPS exposure would result in higher internal concentrations of unconjugated BPs in both serum and gonads compared to BPA [11]. As a well-characterized high-risk EDC, BPA induces particularly severe damage to the male reproductive system through multi-tiered mechanisms. Primarily, it mimics estrogen structurally and functionally by binding to estrogen receptors (ERs), thereby disrupting hypothalamic-pituitary-gonadal (HPG) axis regulation and suppressing testosterone (T) levels [12,13]. Concurrently, BPA also interacts with other hormone receptors (e.g., androgen and thyroid receptors), altering their physiological functions [14,15]. Compelling evidence has confirmed that BPA exposure significantly impairs testicular structure and function, inducing seminiferous tubule atrophy, Sertoli cell damage, abnormal spermatogenic cell morphology, and reduced sperm quality (e.g., increased malformation rate and decreased motility) [16,17,18,19]. Moreover, BPA triggers aberrant testicular cell autophagy, exacerbating oxidative stress and apoptotic cell death, which ultimately leads to spermatogenic dysfunction [20]. Given the critical window of vulnerability in reproductive development (particularly from embryonic to prepubertal stages), early-life BPA exposure may induce irreversible reproductive injury via epigenetic modifications (e.g., DNA methylation, histone acetylation) that disrupt germ cell programmed development [21]. Although certain natural drugs, such as vitamin E (VE), can partially mitigate BPA’s reproductive toxicity, they primarily act through a single antioxidant pathway and may pose potential side effects, thereby limiting their clinical application [22,23]. Consequently, identifying highly effective, low-toxicity natural protectants constitutes a critical research frontier.

Natural products are attracting growing research interest due to their diverse bioactive compounds and relatively low side effects. *Pfaffia glomerata* (Spreng). Pedersen, commonly known as Brazilian ginseng, is a perennial herb belonging to the genus *Pfaffia* in the family Amaranthaceae. In Brazilian traditional medicine, it is known as “Para Tudo” (synonymous with a universal remedy). Possessing a medicinal history spanning over 300 years, it exhibits a broad range of therapeutic applications [24,25,26]. The species is primarily distributed throughout the rainforest regions of South America, including Brazil, Ecuador, and Panama [27]. As a traditional medicinal plant, it contains numerous bioactive compounds demonstrating various pharmacological activities, including antioxidant [28], anti-inflammatory [29], and anti-fatigue effects [30]. In recent years, growing interest in natural products has highlighted the potential therapeutic applications of Pg in reproductive health. Animal studies have demonstrated that Pg significantly elevates serum T levels in orchidectomized rats, promotes spermatogenesis, and alleviates testicular injury induced by chemotherapeutic agents [31]. However, systematic research investigating the protective effects of Pg against BPA-induced reproductive system injury and its underlying mechanisms remains scarce. Therefore, this study aims to utilize network pharmacology to explore the potential mechanisms of action of Pg, followed by in vivo validation of its protective effects against BPA-induced male reproductive toxicity and the associated mechanisms. This work seeks to provide a scientific basis for natural preventive strategies against BPA-induced reproductive toxicity while offering new insights into the medicinal development of Pg.

## 2. Results

### 2.1. Ingredients of Pg

**Figure 1:**
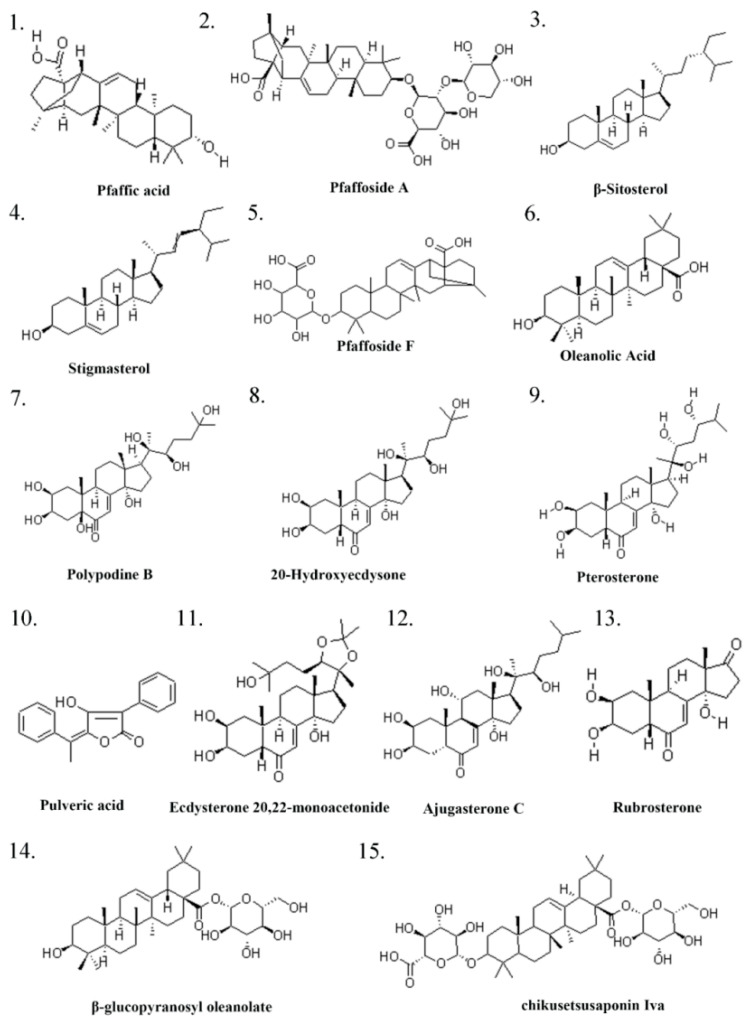
Pg ingredient I.

**Figure 2:**
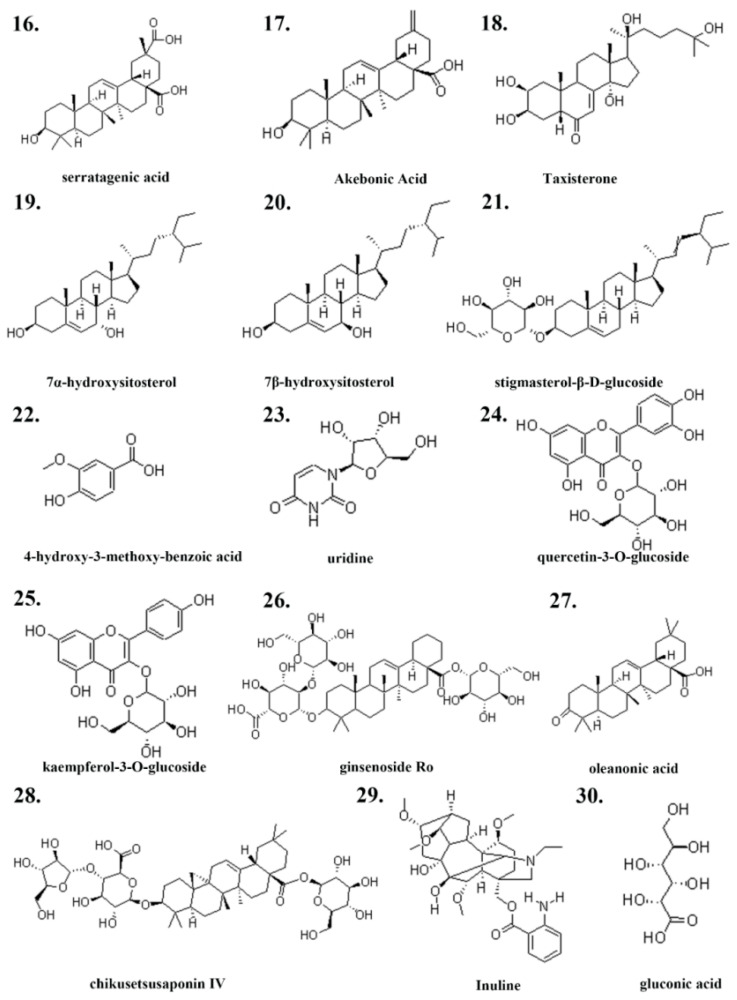
Pg ingredient II.

Screen the primary active ingredients and CAS numbers of Pg from both Chinese and English literature related to Pg, as shown in Table 1 and Figure 1 and Figure 2.

### 2.2. Generation of Drug-Disease Venn Diagram

**Figure 3:**
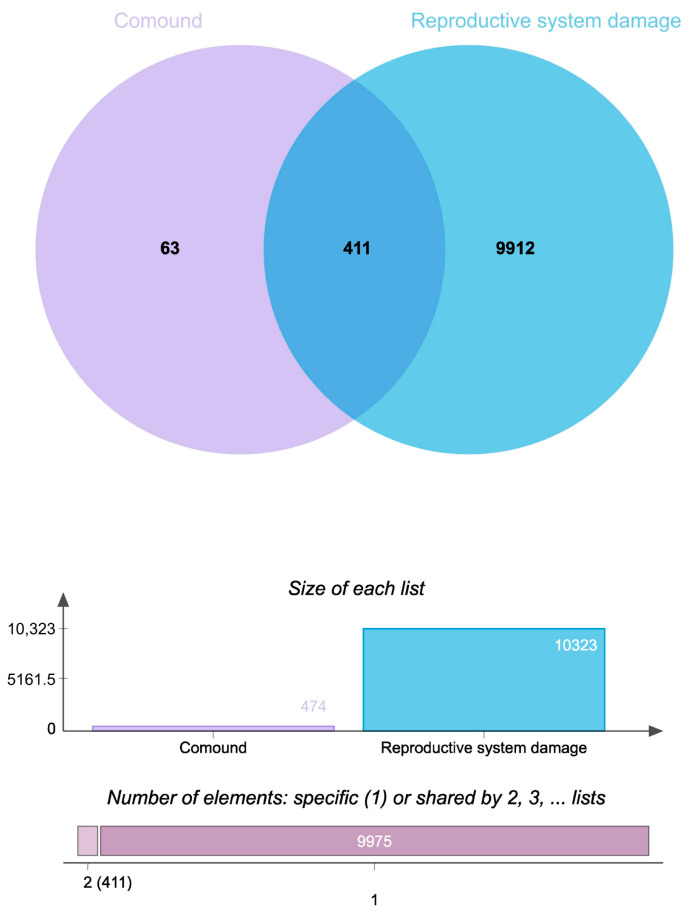
Venn diagram of the overlapping targets between Pg and reproductive system injury.

Database screening identified 10,323 reproductive system injury-associated targets from GeneCards, OMIM, and TTD databases. SwissTargetPrediction analysis (probability > 0) revealed 474 putative compound targets. The intersection between drug target points and disease target points demonstrated 411 overlapping targets, as depicted in Figure 3.

### 2.3. Ingredient-Target-Disease Network Analysis

**Figure 4:**
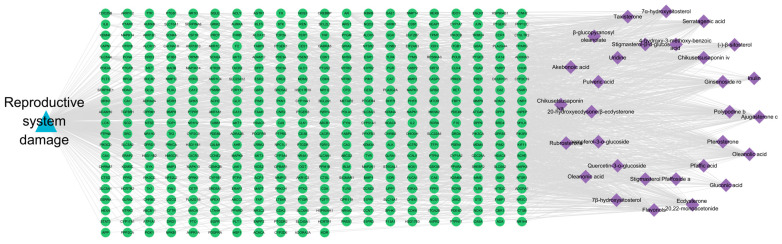
Ingredient-target-disease network diagram.

The constructed network revealed significantly higher edge connectivity for key constituents, including Flavonols, Ginsenoside Ro, Chikusetsusaponin IV, and Oleanonic acid, compared to other components (Table 2). This topological feature suggests that these compounds may function as core mediators of Pg’s therapeutic effects on reproductive system injury. Figure 4 illustrates the network, where the green area denotes targets, the purple area indicates components, and the blue area represents diseases.

### 2.4. PPI Network Analysis

**Figure 5:**
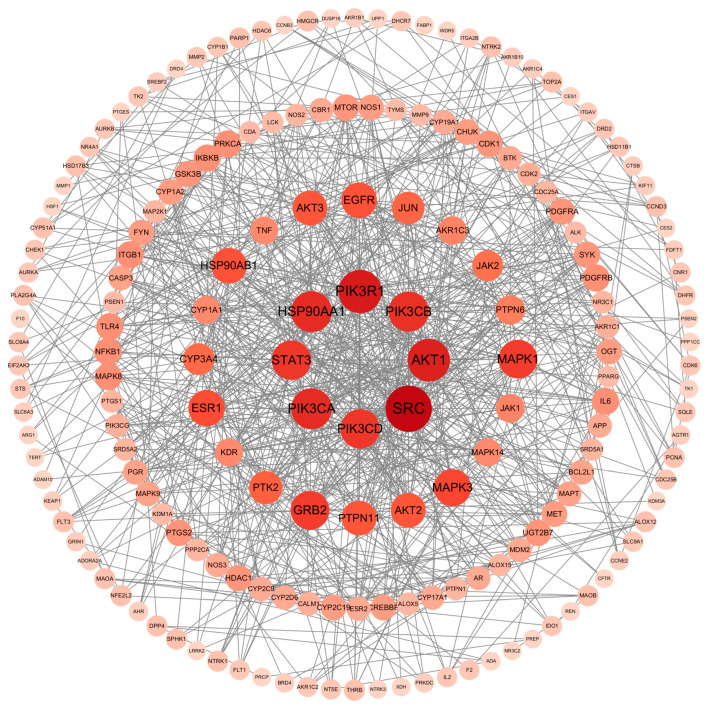
PPI network of the potential targets of Pg for reproductive system injury.

Shared targets were analyzed in the STRING database to generate a PPI network diagram (Figure 5). A network was constructed with a confidence level >0.9 and a degree filter >2, resulting in a final network of 181 nodes and 812 edges following the removal of unconnected nodes. In the protein–protein interaction (PPI) network, each node represents a potential target, and each edge signifies an interaction between two targets. The layout of the nodes is determined by their degree value, which is a key measure of connectivity. Specifically, a node positioned closer to the center of the con-centric circles with a darker color indicates a higher degree value, meaning it interacts with a greater number of other nodes in the network.

**Figure 6:**
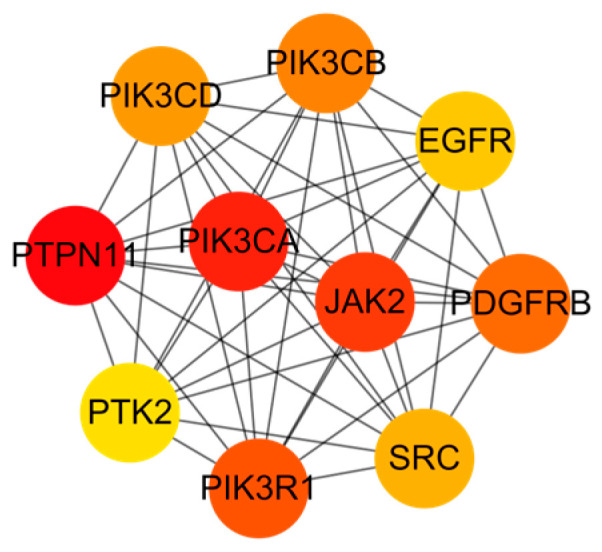
The top 10 potential core targets of Pg for attenuating reproductive system injury.

Meanwhile, using the MCC algorithm, the top ten targets were selected: PTPN11, PIK3CA, JAK2, PIK3R1, PDGFRB, PIK3CB, PIK3CD, SRC, EGFR, and PTK2, as illustrated in Figure 6. These targets functioned as central hubs within the network, indicating their critical role as primary targets for Pg bioactive constituents in mitigating repro-ductive system injury. Furthermore, a substantial portion of these core targets are di-rect components or critical upstream regulators of the PI3K/AKT signaling pathway. Multiple catalytic and regulatory subunits of the PI3K complex itself (PIK3CA, PIK3R1, PIK3CB, PIK3CD) were identified [37,38], alongside key receptor tyrosine kinases (EGFR, PDGFRB) known to activate this pathway [39,40]. The dense connectivity of the core subnetwork further supports the essential function of Pg active components in modulating mechanisms underlying reproductive system impairment, and suggests that the PI3K/AKT signaling pathway may be the core mechanism by which Pg treats reproductive system injuries.

### 2.5. GO/KEGG Enrichment Analysis

**Figure 7:**
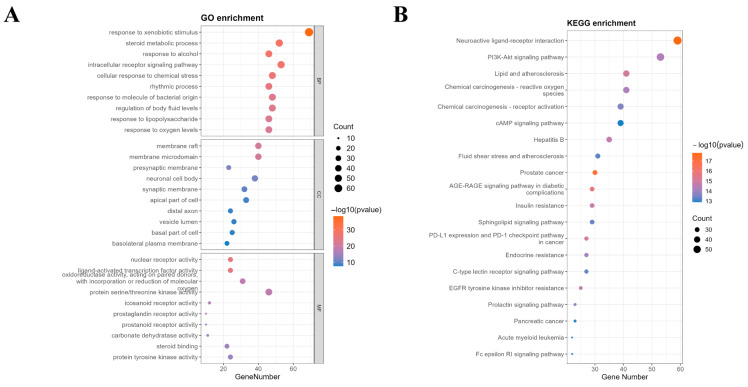
GO and KEGG enrichment analysis of common targets between Pg and reproductive system injury. (**A**) GO enrichment diagram; (**B**) KEGG pathway enrichment diagram.

GO function and KEGG pathway enrichment results are presented in Figure 7. GO enrichment analysis identified a total of 2315 biological processes (BPs), 99 cellular components (CCs), and 238 molecular functions (MFs). Based on the *p*-value, the top 10 entries for each category are detailed in Figure 7A. The BP primarily involved responses to xenobiotic stimuli, steroid metabolic process, and reactions to alcohol. The CC mainly encompassed the membrane raft, membrane microdomains, and presynaptic membranes. The MF primarily included nuclear receptor activity, ligand-activated transcription factor activity, and oxidoreductase activity.

**Figure 8:**
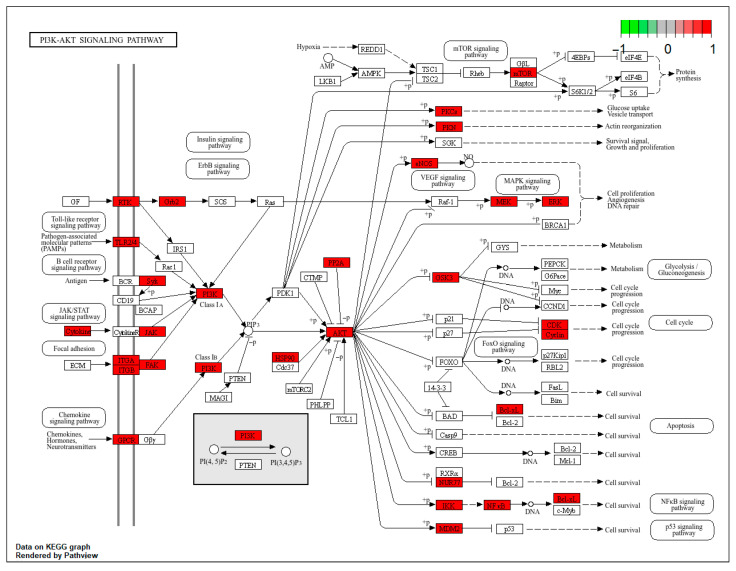
Diagrams of the PI3K-AKT signaling pathway. Highlighting the target that is common to both Pg and reproductive system injury, represented in red color.

To systematically elucidate the mechanisms through which Pg alleviates reproductive system injury, we performed KEGG pathway enrichment analysis on its potential targets. This analysis identified 177 statistically significant pathways (*p* < 0.05). Figure 7B presents a bubble chart of the top 20 pathways ranked by the number of involved genes. Among these, neuroactive ligand–receptor interaction, the PI3K-AKT signaling pathway, and lipid and atherosclerosis were the most prominently enriched. Moreover, several core targets, such as PIK3CA, PIK3CB, PIK3CD, PIK3R1, and EGFR, demonstrated high relevance to the PI3K-AKT signaling pathway, reinforcing its central importance. Given the well-established role of the PI3K-AKT pathway as a master regulator of cell survival, proliferation, and metabolism, and its prominent enrichment in our dataset, we selected it for further mechanistic investigation. Consequently, a schematic diagram illustrating the therapeutic role of Pg against reproductive system injury via targeting the PI3K-AKT pathway is proposed in Figure 8. These findings suggest that Pg components play a role in mitigating reproductive system injury by regulating multiple pathways, including receptor signaling, oxidative stress response, and metabolic homeostasis, and the PI3K-AKT signaling pathway was identified as a hub of particular interest for further mechanistic investigation.

### 2.6. Quantification of Total Saponins in Pg

**Figure 9:**
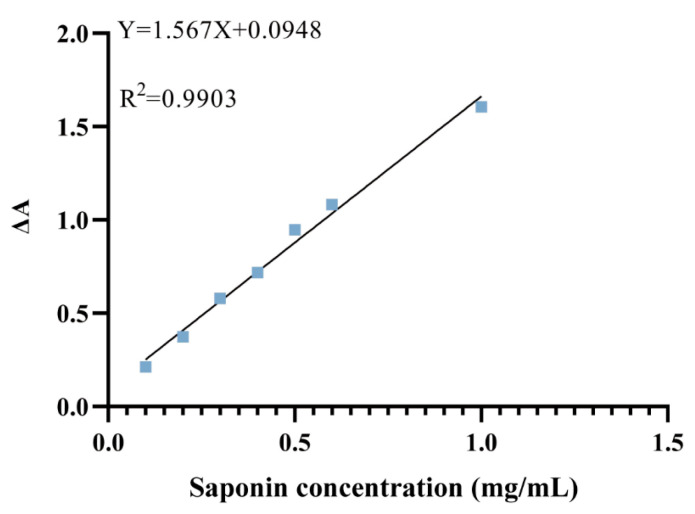
Total Saponin Standard Curve.

The vanillin-perchloric acid colorimetric method yielded a standard curve with a regression equation of y = 1.567x + 0.0948, and an R^2^ value of 0.9903, indicating excellent linearity (Figure 9).

Utilizing the linear regression equation, the extraction yield of Pg saponins was 34.70%, while the total saponin content was 24.26%.

### 2.7. Pg Attenuates BPA-Induced Sperm Abnormalities

**Figure 10:**
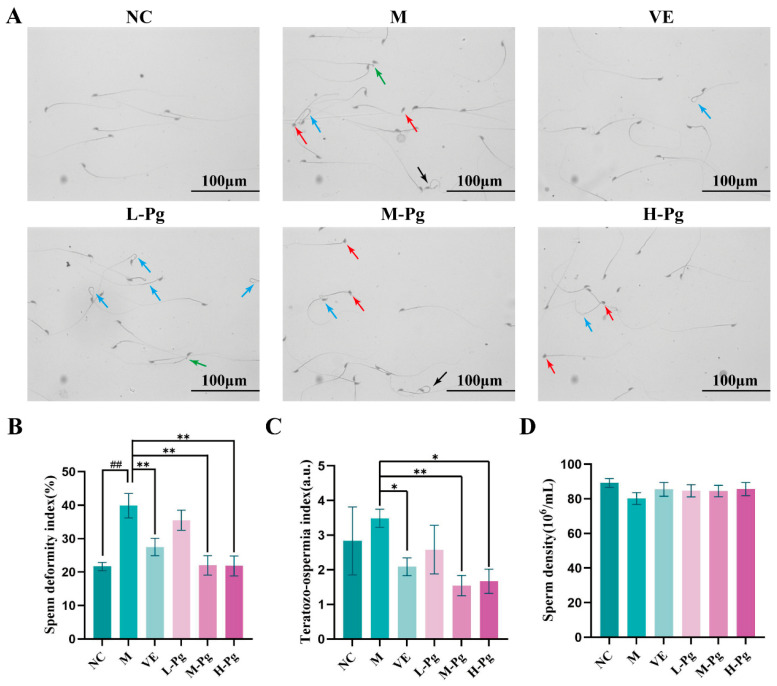
Morphological analysis of sperm rapid modified Papanicolaou staining in different groups. (**A**) Photomicrograph of spermatozoa; (**B**) Spenn deformity index; (**C**) Teratozo-ospermia index; (**D**) Sperm density. All the data are presented as the means ± SDs (*n* = 10); Compared with NC group, ^##^ *p* < 0.01; Compared with M group, * *p* < 0.05, ** *p* < 0.01. Head defects are indicated by red arrows, tail defects by blue arrows, midpiece defects by black arrows, and cytoplasmic droplet retention by green arrows.

Under optical microscopy, sperm morphology in mouse vasa deferentia was examined across groups (Figure 10A). In the M group, there was a significant increase in the incidence of spermatozoa exhibiting head defects, tail defects, midpiece defects, and cytoplasmic droplet retention. The SDI in the M group was significantly higher than that of the NC group (*p* < 0.01), validating successful injury modeling after BPA intervention (Figure 10B). Post-treatment with VE and Pg increased sperm density (Figure 10D) and reduced abnormal sperm counts significantly compared to the M group. Additionally, both the SDI and the TZI in the M-Pg and H-Pg groups showed significant improvement relative to the M group (*p* < 0.05), demonstrating the efficacy of Pg in counteracting BPA-induced spermiodefects (Figure 10C).

### 2.8. Pg Improves BPA-Induced Injury to Testicular Tissue Structure

**Figure 11:**
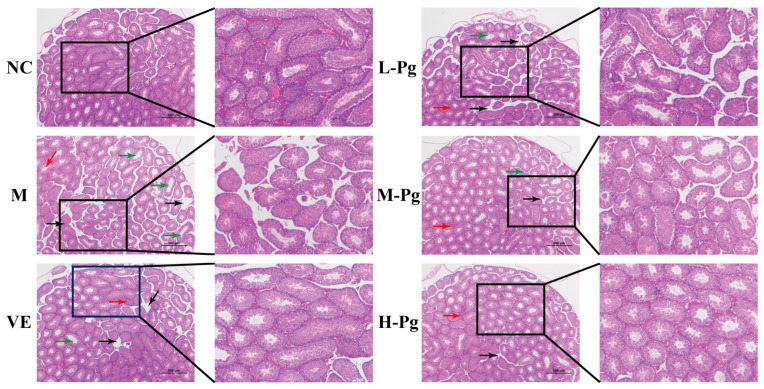
Histological analysis of tesis HE staining in different groups (*n* = 6).

The testes serve as the primary site for sustaining male reproductive function, mediating spermatogenesis and testosterone secretion, which are essential for maintaining androgen-dependent physiology [41]. Histopathological analysis of testicular cross-sections, as demonstrated in the HE staining results (Figure 11), indicates that the testes of the NC group mice exhibit a well-preserved tissue structure, complete seminiferous tubules, and a plentiful presence of Leydig cells, with the spermatogenic epithelium organized systematically. After BPA treatment, the seminiferous tubules in the testicular tissue of the M group mice displayed significant atrophy, characterized by a pronounced reduction in the number of Leydig cells and basal lamina hyperplasia (indicated by red arrows). This induced lumen dilatation in seminiferous tubules (indicated by black arrows). Basement membrane discontinuity led to disorganization of spermatogenic epithelium. Sertoli cells were significantly reduced with cytoplasmic vacuolation, while spermatogenic cells were dislodged from spermatogenic epithelium. Additionally, there was a substantial decrease in the number of mid-to-late-stage spermatids (highlighted by green arrows), indicating BPA-induced severe testicular lesions and disorders in spermatogenesis. Following VE treatment, the severity of testicular lesions improved, accompanied by Leydig cell hyperplasia. This suggests that VE has partial cytoprotective effects on the spermatogenesis process; however, its capacity to mitigate BPA-induced injury to testicular tissue structure appears to be limited. In the L-Pg group, the integrity of the spermatogenic epithelium showed moderate restoration, but the protective effect was weak, and lesions persisted with incomplete remediation. Intervention with moderate to high doses of Pg demonstrated a significant cytoarchitectural protection, characterized by the typical morphology of spermatogenic cells and the restoration of normal numbers of Sertoli cells and Leydig cells. These changes are crucial for the testis to maintain normal male reproductive function. However, long-term exposure to BPA still leads to irreversible damage, such as pathological dilation of the seminiferous tubules and dysregulated secretory activity by some Leydig cells, which persist.

**Figure 12:**
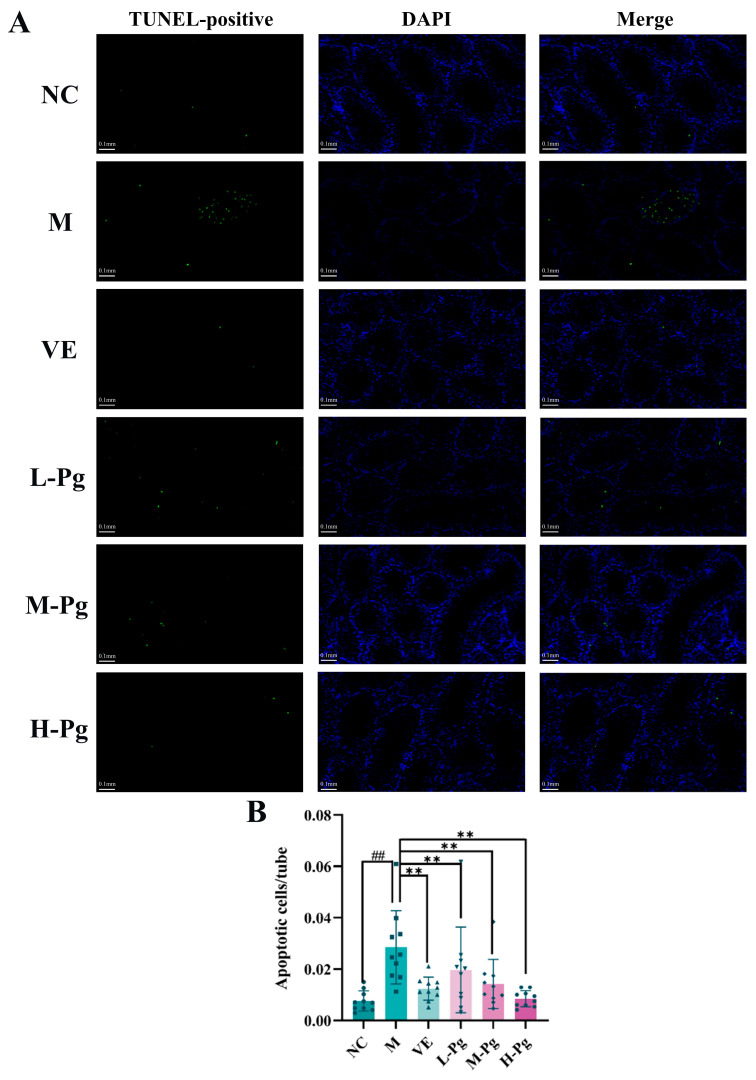
Different groups of TUNEL staining (*n* = 10). (**A**) Representative images of TUNEL staining showing apoptotic cells in testicle tissues from different treatment groups; scale bar: 0.1 mm. (**B**) Quantitative analysis of TUNEL-positive cells in each group. All the data are presented as the means ± SDs (*n* = 10); Compared with NC group, ^##^ *p* < 0.01; Compared with M group, ** *p* < 0.01.

Testicular apoptosis was evaluated by TUNEL assay (Figure 12). DAPI-counterstained nuclei (blue) showed nuclear morphology, while TUNEL-positive nuclei (green) marked apoptotic nuclei. Quantitative analysis demonstrated a significant increase in the number of apoptotic cells in the M group compared to the NC group (*p* < 0.01), indicating that BPA exposure substantially promotes testicular apoptosis. Pg intervention dose-dependently reduced apoptosis, with significantly fewer apoptotic cells per seminiferous tubule compared to the M group (*p* < 0.01). Further analysis indicated that apoptosis primarily occurred in basal compartment spermatogonia and Sertoli cells, suggesting that these two cell types were susceptible to BPA-induced injury and demonstrated significant recovery after Pg administration.

### 2.9. Sex Hormone Panel and Testicular Biomarkers Analysis

**Figure 13:**
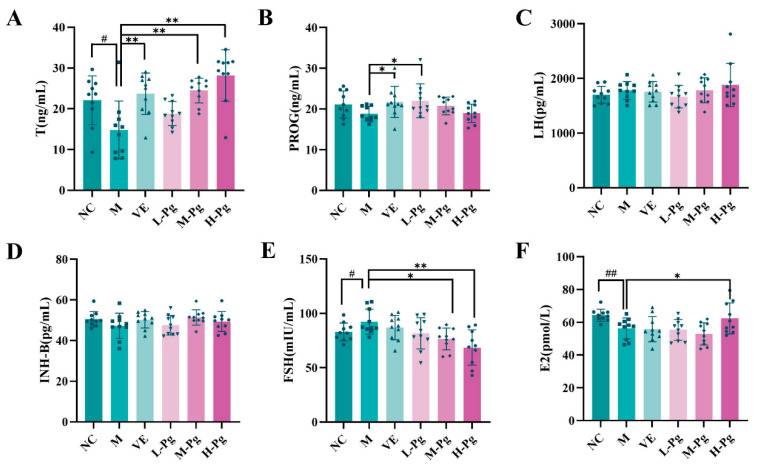
Changes in sex hormone in different groups. (**A**) T; (**B**) PROG; (**C**) LH; (**D**) INH-B; (**E**) FSH; (**F**) E2. All the data are presented as the means ± SDs (*n* = 10); Compared with NC group, ^#^ *p* < 0.05, ^##^ *p* < 0.01; Compared with M group, * *p* < 0.05, ** *p* < 0.01.

The six-hormone panel serves as a crucial indicator for evaluating reproductive system function, providing an objective reflection of the body’s endocrine status and male reproductive health. In Figure 13, the results indicated that BPA exposure significantly impaired Leydig cell function, resulting in a notable decrease in T secretion levels (*p* < 0.05). Serum T levels in the M-Pg and H-Pg groups were significantly elevated compared to the M group (*p* < 0.01), exceeding positive control VE efficacy. Furthermore, FSH levels in the M group were significantly increased compared to the NC group (*p* < 0.05), while Pg treatment induced dose-dependent FSH reduction, with significant regulation in M-Pg and H-Pg groups (*p* < 0.05). This finding may be attributed to FSH-mediated INH-B production and pituitary feedback [42]. Although LH is theoretically capable of regulating T synthesis by stimulating Leydig cells, no significant intergroup differences in LH levels were observed in this study.

Subsequent research has demonstrated that BPA exposure can significantly impair the endocrine function of testicular Sertoli cells, leading to a marked reduction in the secretion of E2 (*p* < 0.01). However, high-dose Pg intervention has been shown to effectively reverse this suppression, with H-Pg group E2 levels significantly exceeding M group (*p* < 0.05). These results suggest that Pg may contribute to enhancing Sertoli cell secretory capacity.

**Figure 14:**
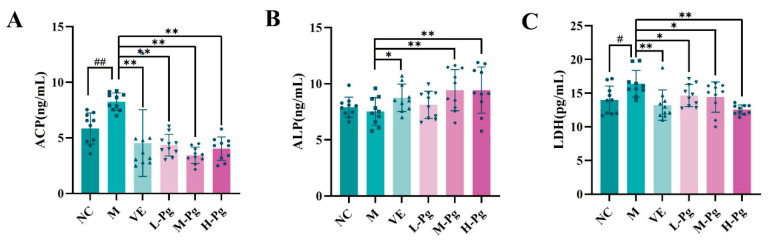
Changes in testicular marker enzymes in different groups. (**A**) ACP; (**B**) ALP; (**C**) LDH. All the data are presented as the means ± SDs (*n* = 10); Compared with NC group, ^#^ *p* < 0.05, ^##^ *p* < 0.01; Compared with M group, * *p* < 0.05, ** *p* < 0.01.

The activity of testicular marker enzymes is essential for sustaining spermatogenesis and serves as a vital indicator of testicular function [43]. ACP is primarily found in the cytoplasm of Sertoli cells [44]. Exposure to BPA resulted in an abnormal elevation of ACP levels in the M groups (Figure 14A), with the seminiferous epithelium exhibiting a significant increase in ACP activity (*p* < 0.01). Both VE and Pg interventions effectively reduced ACP levels (*p* < 0.01), bringing them back within the normal range.

ALP, a specific marker enzyme for germ cells, demonstrates robust activity in both spermatogonia and early-stage primary spermatocytes. Exposure to BPA disrupts the seminiferous epithelium and harms germ cells, leading to a marked reduction in ALP activity [45]. As shown in Figure 14B, ALP activity in the M group declined significantly compared to the NC group following BPA exposure. However, administration of medium and high doses of Pg significantly restored ALP activity (*p* < 0.01).

LDH, an enzyme serving as a marker for cytoplasmic integrity [46], is released upon cell membrane damage and reflects cytoplasmic enzyme activity. Exposure to BPA disrupts the membranes of spermatogenic cells, leading to enzyme leakage and a significant increase in LDH activity. This finding suggests that BPA may impair spermatogenesis and disturb hormone secretion. As shown in Figure 14C, LDH activity was markedly elevated (*p* < 0.05) in the M group compared to the controls. Both VE and Pg treatments effectively reduced LDH activity (*p* < 0.05), with the H-Pg group demonstrating a particularly significant decrease (*p* < 0.01), bringing levels close to normal.

### 2.10. Cell Apoptosis-Related Indicator Testing

**Figure 15:**
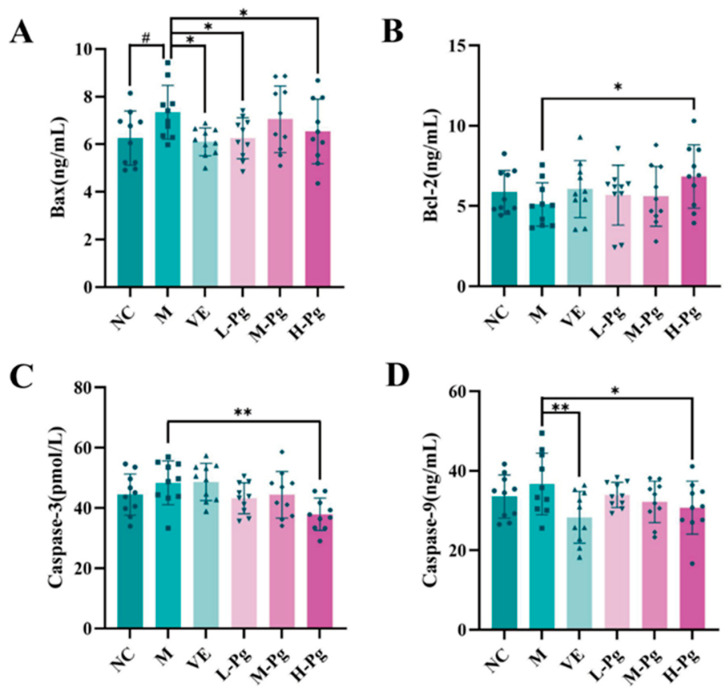
Changes in apoptosis indicators in different groups. (**A**) Bax; (**B**) Bcl-2; (**C**) Caspase-3; (**D**) Caspase-9. All the data are presented as the means ± SDs (*n* = 10); Compared with NC group, ^#^ *p* < 0.05, Compared with M group, * *p* < 0.05, ** *p* < 0.01.

Hormones such as T are vital for normal cellular function and autophagy within the testes. Exposure to BPA can disrupt hormonal balance, potentially impairing the regulation of autophagy [47]. Figure 15 illustrates the expression levels of apoptosis-related proteins, including Bax, Bcl-2, Caspase-3, and Caspase-9, in testicular homogenates across different groups. In comparison to the NC group, the M group exhibited a significantly increased expression of Bax (*p* < 0.05). Although the decrease in anti-apoptotic Bcl-2 was not statistically significant, it demonstrated a downward trend. Notably, high-dose Pg intervention significantly normalized the expression levels of Bax, Bcl-2, Caspase-3, and Caspase-9 compared to the M group (*p* < 0.05). Significantly, the expression of Caspase-3, the key executioner protease in the apoptosis pathway, was markedly reduced (*p* < 0.01). These results suggest that high-dose Pg provides anti-apoptotic protection by upregulating Bcl-2, inhibiting the pro-apoptotic activity of Bax, and blocking the activation cascade of Caspase-9 and Caspase-3.

### 2.11. Pg Attenuates Testicular Injury by Inhibiting Apoptosis via Suppression of the BPA-Induced PI3K/AKT Pathway

**Figure 16:**
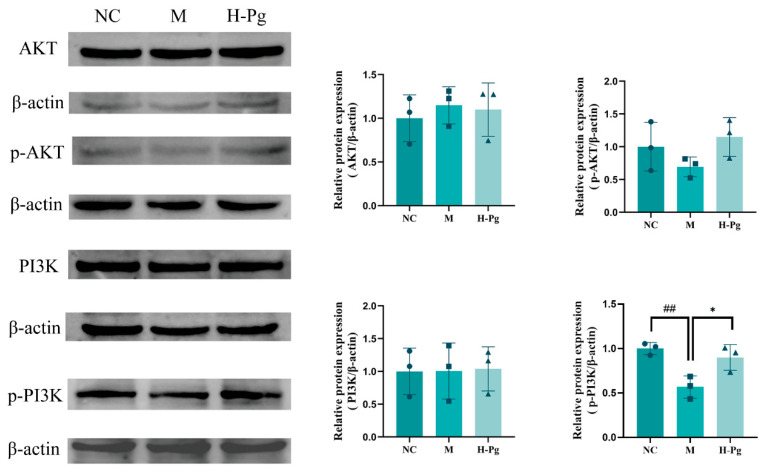
PI3K/AKT pathway-related protein expression in different groups (*n* = 3). Compared with NC group, ^##^ *p* < 0.01; Compared with M group, * *p* < 0.05.

**Figure 17:**
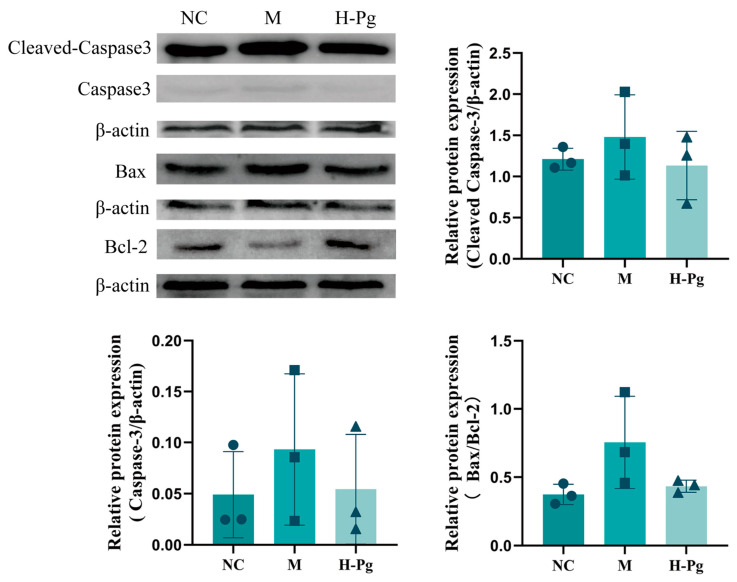
Expression of apoptosis-related proteins in different groups (*n* = 3).

This study investigated the molecular mechanisms underpinning BPA-induced apoptosis in testicular cells by systematically examining the expression levels of key proteins in the PI3K/AKT signalling pathway through Western blot analysis. The expression of eight apoptosis-related proteins in testicular tissue was analyzed: PI3K, p-PI3K, AKT, p-AKT, Caspase-3, Cleaved Caspase-3, Bcl-2, and Bax (Figure 16 and Figure 17). The results indicated that BPA exposure significantly inhibited the activation of the PI3K/AKT pathway, as evidenced by the reduced levels of p-PI3K and p-AKT in the M group. Consistent with PI3K/AKT pathway inhibition, corresponding changes were observed in downstream apoptosis-related proteins: Cleaved Caspase-3 and Bax expressions were elevated, while Bcl-2 levels decreased. In contrast, high-dose Pg effectively reversed these BPA-induced changes, leading to increased expression of p-PI3K and p-AKT, downregulation of Cleaved Caspase-3 and Bax, and upregulation of Bcl-2. These findings suggest that Pg may provide anti-apoptotic protection by activating the PI3K/AKT pathway and modulating the expression of key downstream apoptosis regulators.

## 3. Discussion

Numerous in vitro and in vivo studies have confirmed that BPA and its analogues exert significant toxic effects on male reproductive function and sperm quality, with oxidative stress and excessive ROS production constituting the primary pathogenic mechanism [48,49]. Sperm quality is a key indicator for assessing male fertility [50], and alterations in sperm quality are closely linked to the regulation of the dynamic balance between cell proliferation, differentiation, and apoptosis during spermatogenesis. This study explored the mechanism by which BPA induces excessive apoptosis in testicular cells, leading to reproductive system dysfunction from both network pharmacology and experimental validation perspectives, and assessed the potential of Pg extract to mitigate such damage by activating the PI3K/AKT signaling pathway.

Network pharmacology findings suggest that the active components of Pg operate via a multi-target, multi-pathway strategy. These components may enhance recovery from BPA-induced reproductive system injury through multiple mechanisms, including regulating steroid metabolism, enhancing antioxidant capacity, and modulating signaling pathways (such as the PI3K/AKT, Fc epsilon RI, and cAMP signaling pathways). These pathways are closely linked to germ cell proliferation, apoptosis, and inflammatory responses, providing a theoretical foundation for understanding how Pg enhances reproductive recovery. KEGG enrichment analysis revealed that both the number of genes enriched in the PI3K/AKT pathway, and its significance *p*-value ranked among the highest. Significant crosstalk with other enriched pathways (such as AGE-RAGE) was observed, indicating that the PI3K/AKT pathway plays a dominant role in the Pg target network and exhibits cross-regulatory effects [51]. Given that the PI3K/AKT pathway is a crucial regulator of essential biological processes such as apoptosis [52], and its dysfunction is implicated in BPA-induced reproductive system injury [53], it is suggested that Pg may mitigate germ cell damage caused by excessive apoptosis by suppressing overactivation of the PI3K/AKT pathway.

Animal studies indicate that prolonged BPA exposure can elevate SDI and TZI levels, impair testicular histoarchitecture, and cause significant dysregulation of sex hormones such as E2 and FSH, along with testicular marker enzymes like ACP. Currently, no adequate and reliable therapeutic drugs exist to address BPA-induced injury to the reproductive system. VE, a classic liposoluble antioxidant [22], can provide targeted protective effects by modulating pathological apoptosis and suppressing the lipid peroxidation chain reaction triggered by ROS-mediated oxidative stress signaling. Nonetheless, its sole antioxidant mechanism cannot effectively reverse or repair the multiple toxic cascade reactions triggered by BPA. This study demonstrated that VE intervention significantly mitigated BPA-induced reproductive system injury, as evidenced by reduced SDI and TZI, amelioration of defects in seminiferous tubule lumens, and decreased testicular tissue apoptosis. However, VE failed to significantly restore sex hormone levels, testicular marker enzyme activity, or testicular histoarchitectural integrity.

Compared to VE, Pg demonstrates superior efficacy in attenuating sperm defects, restoring testicular histoarchitecture, regulating sex hormones, and modulating testicular marker enzyme activity. In recent years, a variety of natural products have been extensively investigated and demonstrated potential in attenuating BPA-induced reproductive injury. For instance, *Cistanche tubulosa* (Schenk) R. wight and its active constituent, echinacoside, have been shown to significantly attenuate BPA-induced decline in sperm quality and testicular toxicity in rats by upregulating steroidogenic enzymes [54]. Similarly, p-coumaric acid has been confirmed to effectively mitigate testicular inflammation and oxidative stress caused by BPA, with network pharmacology analyses suggesting the involvement of the KEAP1/NRF2/ARE pathway in its protective mechanism [55]. Additionally, Eruca sativa leaves aqueous extracts have exhibited protective effects against BPA-induced testicular injury, likely attributable to the antioxidant activity of their phenolic constituents, though the precise mechanisms remain to be fully elucidated [56]. While most existing research has focused on the antioxidant and anti-inflammatory properties of natural products, the specific signaling pathways involved are often not clearly delineated. More importantly, few studies have deeply linked such protective effects to the critical process of inhibiting germ cell apoptosis

Its in vivo anti-apoptotic capacity is notably pronounced. In vivo pharmacodynamic studies indicate that Pg is superior to VE in attenuating sperm abnormalities. Following medium-to-high dose Pg intervention, defects in sperm heads and curled tails were significantly ameliorated, and SDI returned to baseline levels. As the testes are the primary site of sperm production, sperm quality is closely linked to the testicular histoarchitectural integrity. BPA-induced testicular injury impairs spermatogenesis and consequently disrupts normal sperm formation [57]. Testicular HE staining results demonstrate that Pg exerts vital protection against histoarchitectural injury. This was evidenced by significant attenuation of BPA-induced seminiferous tubule atrophy, preservation of basement membrane integrity, and rectification of morphological abnormalities in spermatogenic cells at all developmental stages. Pg treatment also restored Sertoli and Leydig cell populations, promoted proliferation of mid-to-late-stage spermatogenic cells, and protected spermatogenesis. As the primary site of T production and hormonal regulation, testicular function critically influences male reproductive health status. Testicular function is regulated by the hypothalamic-pituitary-gonadal (HPG) axis. Gonadotropin-releasing hormone (GnRH) stimulates the anterior pituitary to secrete LH and FSH. These hormones bind receptors on Leydig and Sertoli cells, promoting T production and spermatogenesis [57]. This study indicates that Pg can dose-dependently increase serum T levels while decreasing FSH levels, suggesting mitigation of both BPA-induced testicular dysfunction and aberrant T secretion. Importantly, LH stimulates T biosynthesis in Leydig cells via G protein-coupled seven-transmembrane receptors [58]. However, LH levels did not differ significantly between groups in this study, possibly due to the specificity of LH-activated downstream signaling pathways. Testicular marker enzymes are essential for both spermatogenesis and testicular homeostasis. Among these enzymes, ACP and ALP are well-established biomarkers for primordial germ cells [59]. Additionally, LDH plays a crucial role in sperm functionality by facilitating flagellar glycolysis and energy metabolism [60]. Moderate-to-high doses of Pg normalized these marker enzyme levels, indicating protection against BPA-induced germ cell toxicity through the regulation of hormonal balance and the activities of enzymes such as ACP and ALP.

The pharmacodynamic experiments demonstrated that Pg significantly attenuates BPA-induced testicular cell injury by preserving endocrine balance and enhancing male reproductive function. This effect is primarily mediated through anti-apoptotic mechanisms. Since apoptosis of testicular tissue cells is a critical aspect of BPA toxicity, this study utilized TUNEL staining to evaluate the therapeutic effects of Pg on BPA-induced harm. The TUNEL assay utilizes terminal deoxynucleotidyl transferase (TdT) to catalyze the specific binding of fluorescently labeled deoxyribonucleotides to the 3’-OH termini of DNA double-strand breaks (DSBs), thereby enabling the in situ quantification of apoptotic cells [61]. This method is the current gold standard for apoptosis detection. BPA exposure induced abundant TUNEL-positive nuclei (green fluorescence) in testicular tissues of the M-group, indicating extensive apoptosis. However, with an increase in Pg dosage, the percentage of apoptotic cells markedly reduced, and the anti-apoptotic ability of the H-Pg group exhibited superior anti-apoptotic efficacy compared to the VE positive control. These results suggest that the in vivo anti-BPA effects of high-dose Pg correlate strongly with the suppression of apoptosis, indicating that Pg exhibits considerable anti-apoptotic properties and can increase the expression levels of anti-apoptotic proteins.

TUNEL staining results further confirm that apoptosis is a primary mechanism behind BPA-induced reproductive toxicity, while Pg has notable protective effects against BPA-related injury to the reproductive system. The Bcl-2 protein family, critical regulators of apoptosis, modulates the mitochondrial intrinsic pathway through pro-apoptotic (e.g., Bax) and anti-apoptotic members (e.g., Bcl-2) [62,63]. Anti-apoptotic Bcl-2 localizes to mitochondrial, endoplasmic reticulum, and nuclear membranes, inhibiting cytochrome c release and Caspase-3 activation [64,65]. The pro-apoptotic protein Bax antagonizes the function of Bcl-2 by forming a heterodimer with it [66]. Therefore, the dynamic balance between Bcl-2 and Bax serves as the core molecular switch regulating cell apoptosis and survival. Caspase-3, as a key protein in the execution phase of apoptosis, directly reflects the degree of cell apoptosis [67,68]. Western Blot analysis revealed that high-dose Pg downregulated pro-apoptotic effectors, such as cleaved Caspase-3 and Bax, while upregulating the anti-apoptotic protein Bcl-2 expression, thereby restoring the physiological Bax/Bcl-2 ratio. This suggests that Pg attenuates BPA-induced reproductive injury by suppressing pathological apoptosis. Thus, the Bcl-2/Bax equilibrium serves as a key molecular switch that regulates cellular apoptosis and survival decisions. Cleaved Caspase-3 executes terminal apoptosis and directly quantifies the progression of apoptosis. Consistent with this, high-dose Pg downregulated testicular pro-apoptotic mediators, such as cleaved Caspase-3 and Bax, while upregulating Bcl-2 expression. These findings confirm that Pg mitigates BPA-induced reproductive injury through apoptosis inhibition.

Our network pharmacology analysis indicates that the PI3K/AKT signaling pathway may be a potential mechanism through which Pg attenuates BPA-induced reproductive system injury. The PI3K/AKT pathway is essential for male reproduction, as it regulates spermatogonial proliferation and differentiation, as well as somatic cell function during spermatogenesis [69]. Additionally, it maintains Sertoli cell redox homeostasis and metabolic activity, which are critical for supporting spermiogenesis. Inhibition of the PI3K/AKT pathway can lead to decreased sperm motility, increased apoptosis, and oxidative DNA damage [70]. Mechanistically, activation of AKT by PI3K promotes the dissociation of Bcl-2 from phospho-Bad, allowing Bcl-2 to inhibit apoptosis and suppress downstream effectors such as Caspase-3 [71]. To further investigate whether Pg affects the PI3K/AKT signaling pathway, we conducted Western Blot experiments. The results demonstrated that Pg reverses the BPA-induced inhibition of the PI3K/AKT pathway, suggesting that Pg may improve BPA-induced reproductive system injury by activating this signaling pathway. A critical direction for future research is to investigate how Pg mitigates BPA-induced damage and whether its mechanisms can be linked to the regulation of the PI3K/AKT pathway, as well as its association with oxidative stress and ferroptosis [20,72]. Such studies will not only provide new mechanistic insights into the protective effects of Pg but also strongly affirm its therapeutic potential as a natural compound that acts through multiple targets and pathways.

Furthermore, while current research on natural interventions against BPA-induced reproductive toxicity has predominantly centered on phenolic compounds, the principal bioactive constituents of Pg are triterpenoids and their saponins [73]. This distinctive composition offers novel perspectives and experimental evidence for the development of natural products with unique structures and mechanisms of action. Beyond its efficacy, the translational relevance of Pg is supported by its historical use and a favorable safety profile. Pg has a longstanding history of use in traditional Brazilian medicine as a tonic for managing sexual dysfunction [29,74], which provides initial evidence of its tolerability in humans. Our toxicity assessment also offers preliminary experimental data to support its safety. The median lethal dose (LD_50_) of the Pg extract was determined to be greater than 8 g/kg, and no mortality was observed even at the maximum tolerated dose of 24 g/kg, although transient adverse effects such as tremors were noted at this highest dose. The wide margin between the effective and toxic doses suggests a favorable short-term safety profile for Pg. Therefore, we propose that future work address its chronic toxicity, pharmacokinetics, and quality evaluation. These steps are crucial to translate Pg into a practical intervention for reproductive health. In conclusion, our study validates the therapeutic effects of Pg against BPA-induced reproductive injury and provides a theoretical foundation for the development of new natural drugs or health supplements.

## 4. Materials and Methods

### 4.1. Bioinformatics Analysis

#### 4.1.1. Acquisition of Pg Bioactive Constituents and Target Prediction

Literature on Pg (e.g., “*Pfaffia glomerata*” and “*Pfaffia paniculata*”) was retrieved from multilingual databases (including Web of Science, PubMed, and CNKI). The primary bioactive constituents and their corresponding CAS numbers were identified. Compound-related targets were predicted using the SwissTargetPrediction database (probability threshold > 0) with duplicate entries removed.

#### 4.1.2. Screening for Reproductive System Injury Targets

Disease targets were acquired by searching GeneCards and the Therapeutic Target Database (TTD) with “Reproductive system injury” as the query term. After removing duplicates, the final set of targets was compiled.

#### 4.1.3. Construction of “Component-Target-Disease” Network

The intersection between Pg constituent targets and reproductive system injury targets was identified and visualized via a Venn diagram. Overlapping targets representing potential therapeutic targets were used to generate an Excel mapping file. Using Cytoscape 3.8.2, a component-target-disease network was constructed and subjected to topology analysis. Core constituents were identified based on Degree, Betweenness Centrality, and Closeness Centrality.

#### 4.1.4. Protein–Protein Interaction (PPI) Network Construction and Core Target Identification

The shared targets were imported into the STRING database, specifying Homo sapiens as the species and setting a minimum confidence score of greater than 0.9. Disconnected nodes were hidden to construct a PPI network of the shared targets between Pg and reproductive system injury. The PPI network was exported as a TSV file and analyzed in Cytoscape 3.8.2. The top 10 core targets were ranked using the Maximal Clique Centrality (MCC) algorithm.

#### 4.1.5. Functional Enrichment Analysis

To elucidate the potential biological functions and pathways of the shared targets, functional enrichment analysis was performed. GO enrichment analysis and KEGG pathway enrichment analysis were conducted using the clusterProfiler package in R language. Bio-enrichment results for these genes, encompassing molecular functions (MF), cellular components (CC), biological processes (BP), and signaling pathways, were obtained through this process. Significantly enriched terms were selected with a significance threshold of an adjusted *p*-value < 0.05 and then ranked by their *p*-value and gene number in order. In this study, we selected the top 10 GO terms and top 20 KEGG pathways and utilized bubble charts to visualize the results. The schematic diagram of the PI3K-AKT signaling pathway was adapted from the KEGG database (https://www.genome.jp/kegg/ accessed on 2 May 2024).

### 4.2. Experimental Materials and Instruments

Pg, purchased from Ningbo Fafeiya Food Co., Ltd. (Ningbo, China), were authenticated by Prof. Haitao Li at the Yunnan Branch of the Institute of Medicinal Plant Development, Chinese Academy of Medical Sciences (Yunnan, China). BPA was obtained from J&K Scientific Ltd. (Beijing, China). VE and Rainbow 180 Broad Range Protein Marker were sourced from Solarbio Science & Technology Co., Ltd. (Beijing, China). Tissue fixation solution was purchased from Servicebio Biotechnology Co., Ltd. (Wuhan, China). Absolute ethanol and xylene were acquired from Sinopharm Chemical Reagent Co., Ltd. (Shanghai, China). Rapid Sperm Staining Kit was obtained from Nanjing Jiancheng Bioengineering Institute (Nanjing, China). The following enzyme-linked immunosorbent assay (ELISA) kits were procured from Shanghai Enzyme-linked Biotechnology Co., Ltd. (Shanghai, China): Mouse ACP, ALP, LDH, Luteinizing Hormone (LH), Inhibin B (INHB), Estradiol (E2), Testosterone (T), Progesterone (PROG), Follicle-Stimulating Hormone (FSH), Bcl-2-associated X protein (Bax), B-cell lymphoma 2 (Bcl-2), Caspase 9 Activity Assay Kit, and Caspase 3 Activity Assay Kit. The BCA Protein Assay Kit was purchased from Beyotime Biotechnology (Shanghai, China). The following reagents were sourced from ComWin Biotech Co., Ltd. (Beijing, China): SDS-PAGE Loading Buffer, Tris-Glycine SDS Running Buffer, TBST Buffer, Tris-Glycine Transfer Buffer, SDS-PAGE Gel Preparation Kit, and Enhanced Chemiluminescence (ECL) Detection Kit. Primary antibodies against Bax, Bcl-2, Caspase-3/Cleaved Caspase-3, PI3K, AKT, and p-AKT were obtained from Wanleibio Co., Ltd. (Shenyang, China). Antibody against p-PI3K was purchased from Bioss Antibodies (Beijing, China). Antibody against β-actin was acquired from Qinke Biotechnology Co., Ltd. (Jiangsu, China). Goat Anti-Rabbit IgG H&L (HRP) secondary antibody was obtained from Abclonal Technology Co., Ltd. (Wuhan, China). All other chemical reagents used were of analytical grade and obtained from local suppliers.

Leica TCS-SP laser scanning confocal microscope (Leica Microsystems, Heidelberg, Germany); BDS400 inverted biological microscope (Aote Optical Instruments Co., Ltd., Guangzhou, China); 5424R high-speed refrigerated centrifuge (Eppendorf AG, Hamburg, Germany); OSE-Y50 tissue homogenizer (Tiangen Biotech Co., Ltd., Shanghai, China); QW-YC-24C rocking shaker (Qiwei Instruments Co., Ltd., Hangzhou, China).

### 4.3. Preparation and Content Determination of Pg Extract

Powdered Pg (20 g) was extracted twice with 50% ethanol (solid-to-solvent ratio 1:20, *w*/*v*) under reflux for 1 h per extraction. The combined filtrates were decolorized with 2% (*w*/*w*) activated charcoal for 2 h, vacuum-filtered, concentrated under reduced pressure, and lyophilized to obtain the Pg extract.

A 5% (*w*/*v*) vanillin-glacial acetic acid solution was used as the reagent. A stock solution of ginsenoside Re (1 mg/mL) was prepared. Aliquots (0.1–0.6 mL) of the stock solution were diluted to 1 mL with methanol to prepare standard solutions. Each standard solution (0.5 mL) was transferred to a 1.5 mL microcentrifuge tube and evaporated to dryness at 70 °C. The residue was reacted with 0.2 mL vanillin reagent and 0.8 mL of perchloric acid at 55 °C for 20 min. The reaction mixture (200 µL) was diluted with 1 mL glacial acetic acid, and absorbance was measured at 589 nm (25 °C). A standard curve was generated by plotting absorbance (y) versus ginsenoside Re concentration (x, mg/mL), and the regression equation was determined.

### 4.4. Animals and Grouping

Healthy male ICR mice (SPF grade, 6–8 weeks old) were obtained from Beijing Huafukang Bioscience Co., Ltd. (Beijing, China, License No.: SCXK (Jing) 2024-0003). All procedures were approved by the Animal Ethics Committee of the Institute of Medicinal Plant Development, Chinese Academy of Medical Sciences (approval number: SLXD-20240516013) and conducted in accordance with relevant guidelines. Mice were acclimated for 7 days under controlled conditions: a temperature of 22 ± 2 °C, a humidity of 55 ± 15%, and a 12 h light/dark cycle. All mice were provided a standard diet ad libitum and acclimated for one week before the experiment.

**Figure 18:**
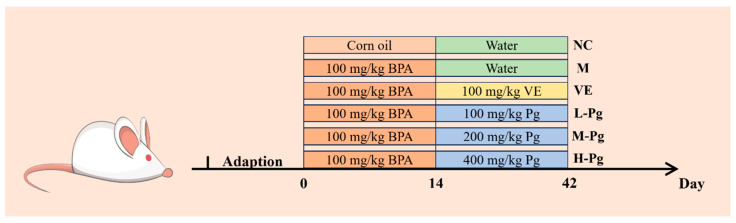
Grouping of animal experiments.

Following acclimatization, 60 male ICR mice (20 ± 2 g) were randomly divided into 6 groups (n = 10/group): normal control (NC) group, model (M) group, VE group, and Pg different doses groups, low-dose (L-Pg) group, medium-dose (M-Pg) group, and high-dose (H-Pg) group. To induce reproductive injury, all groups except the NC group received bisphenol A (BPA) by daily gavage at a dose of 100 mg/kg body weight for 14 consecutive days. Meanwhile, mice in the NC group were administered the corresponding vehicles daily via oral gavage. Starting from day 15, mice in the VE group were orally administered 100 mg/kg/day·bw VE, and mice in the L-Pg, M-Pg, and H-Pg groups received Pg at 100, 200, and 400 mg/kg/day, respectively, via oral gavage for 28 days. Mice in the NC and M groups were daily gavaged with equal volume of the corresponding vehicles. The dosing regimen was determined based on preliminary investigations. The doses of BPA, VE, and Pg were determined based on previously published literature and preliminary experimental results (Supplementary Figure S1 and Table S3). The experimental protocol was illustrated in Figure 18.

Mice had ad libitum access to food and water, with body weights monitored every 48 h. On day 42, mice were fasted overnight (water ad libitum) and reweighed. Anesthesia was induced by intraperitoneal injection of freshly prepared 2,2,2-tribromoethanol (250 mg/kg, 1.25% *w*/*v* in 0.9% saline).

Post-anesthesia procedures comprised: Anogenital distance (AGD) measurement, blood collection via retro-orbital puncture for serum isolation, vas deferens excision for semen collection, and bilateral testis and epididymis excision. Left testes were fixed in 4% paraformaldehyde (PFA) tissue fixative, while right testes were homogenized to prepare a 10% (*w*/*v*) tissue homogenate. All samples were stored at −80 °C until analysis.

### 4.5. Sperm Morphology Assessment

Sperm analysis was conducted according to the WHO Laboratory Manual for the Examination and Processing of Human Semen, Sixth Edition (established by the World Health Organization) [75].

Vasa deferentia were dissected and flushed with 100 μL of pre-warmed physiological saline. Sperm suspensions were incubated at 37 °C for 10 min to allow dispersion. Concentration was assessed using a hemocytometer, counting sperm in four randomized fields per sample under phase-contrast microscopy and calculating the average. Sperm smears were prepared from five randomly selected mice per group. For each mouse, 200 clearly distinguishable spermatozoa were evaluated on the stained smear. Morphologically normal and abnormal spermatozoa were quantified, with abnormal sperm further classified by defect localization into head defects (e.g., amorphous, vacuolated), midpiece defects (e.g., bent, cytoplasmic droplet retention), and tail defects (e.g., coiled, double tail).

Total Number of Defects (TND) = Number of sperm with head defects + Number of sperm with midpiece defects + Number of sperm with tail defects

Sperm Deformity Index (SDI) = TND/Total number of spermatozoa examined

Teratozoospermia Index (TZI) = TND/Number of abnormal spermatozoa

### 4.6. Histopathological Observation

Testicular tissues were fixed in 4% paraformaldehyde, dehydrated through a graded ethanol series, embedded in paraffin, and sectioned at 5 μm thickness using a rotary microtome. Sections were deparaffinized for 10 min and rehydrated through descending ethanol concentrations (100%, 95%, 85%, and 75%) for 5 min each. Following a brief rinse in distilled water (1 min), sections were stained with hematoxylin for 5 min and counterstained with eosin solution for 1–2 s. Sections were then rapidly dehydrated through absolute ethanol (1–2 min) and cleared in xylene. Finally, the sections were examined microscopically before mounting.

### 4.7. TUNEL Staining

Apoptosis in testicular tissues was assessed using the Terminal deoxynucleotidyl transferase dUTP Nick-End Labeling (TUNEL) assay. Staining was performed strictly according to the manufacturer’s protocol of the In Situ Cell Death Detection Kit. The number of TUNEL-positive cells within individual seminiferous tubules was quantified. Micrographs were captured using a Leica TCS-SP laser scanning confocal microscope (Leica, Wetzlar, Germany).

### 4.8. Six-Item Sex Hormone Panel

Serum aliquots were processed and diluted according to the manufacturer-specific protocols provided with the respective ELISA kits. The absorbance was measured at 450 nm using a microplate reader. Standard curves for each hormone were generated by plotting the absorbance values (*Y*-axis) against the corresponding concentrations (*X*-axis) of serially diluted hormone standards. The concentrations of T, PROG, LH, INH-B, FSH, and E2 in the serum samples were then determined based on the generated standard curves and in strict accordance with the manufacturers’ instructions.

### 4.9. Measurement of Apoptosis

Testicular tissue samples (10 mg) were homogenized in 100 μL PBS using a mechanical homogenizer. The homogenate was then centrifuged at 3000 rpm for 10 min to collect the supernatant. Expression levels of apoptosis-related proteins (Bax, Bcl-2, Caspase-3, and Caspase-9) in the testicular homogenate supernatant were quantified using commercial assay kits according to the manufacturer’s protocols.

### 4.10. Protein Extraction and Western Blot

Testicular tissue samples (10 mg) were homogenized in 100 μL of ice-cold RIPA lysis buffer supplemented with protease inhibitor PMSF and phosphatase inhibitors. Homogenates were subsequently centrifuged at 12,000 rpm for 5 min at 4 °C, and supernatants collected. Protein concentrations were quantified using a BCA assay kit. Samples were mixed with SDS-PAGE loading buffer, denatured at 100 °C for 10 min, and briefly centrifuged. Equal protein amounts were separated by electrophoresis on 12% SDS-polyacrylamide gels and subsequently transferred to PVDF membranes. Membranes were blocked with 5% non-fat milk/TBST for 2 h at room temperature, then incubated overnight at 4 °C with the following primary antibodies: anti-PI3K (1:500), anti-p-PI3K (1:500), anti-AKT (1:500), anti-p-AKT (1:500), anti-Bax (1:500), anti-Bcl-2 (1:500), anti-cleaved Caspase 3 (1:500), with anti-β-actin (1:1500) as loading control. After extensive washing with TBST (3 times, 10 min each), membranes were incubated with HRP-conjugated goat anti-rabbit IgG for 1 h at room temperature, washed again thoroughly with TBST (3 times, 10 min each). Protein bands were visualized by incubating membranes with ECL substrate, and chemiluminescent signals were captured using an imaging system. Band intensities were normalized to β-actin and quantified with ImageJ 1.54p (National Institutes of Health, Bethesda, MD, USA).

### 4.11. Data Analysis

Using SPSS 18.0 statistical software for experimental data analysis and organization, the differences between the experimental groups were determined by one-way ANOVA and Tukey’s post hoc test. Data are expressed as mean ± standard deviation (SD). Statistical analyses and data visualizations were conducted with GraphPad Prism 9 (GraphPad Software, Inc., San Diego, CA, USA). All differences were considered to be statistically significant at *p* < 0.05 (* *p* < 0.05 and ** *p* < 0.01).

## 5. Conclusions

In summary, Pg exerts anti-apoptotic effects by activating the PI3K/AKT signaling pathway, thereby mitigating BPA-induced reproductive system injury. This protective effect was evidenced by a significant reduction in the release of pro-apoptotic factors and the expression of pro-apoptotic proteins, thus confirming the predictions of network pharmacology.

## Figures and Tables

**Table 1:** Pg ingredient list.

Serial Number	Ingredient Name	CAS Number	References
1	Pfaffic acid	86432-14-6	[32]
2	Pfaffoside A	90745-17-8	[32]
3	(-)-β-Sitosterol	83-46-5	[32]
4	Stigmasterol	83-48-7	[32]
5	Flavonols	577-85-5	[32]
6	Oleanolic Acid	508-02-1	[33]
7	Polypodine B	18069-14-2	[32]
8	20-Hydroxyecdysone/β-ecdysterone	5289-74-7	[34]
9	Pterosterone	18089-44-6	[34]
10	Pulveric acid	26548-70-9	[32]
11	Ecdysterone 20,22-monoacetonide	22798-96-5	[32]
12	Ajugasterone C	23044-80-6	[32]
13	Rubrosterone	19466-41-2	[34]
14	β-glucopyranosyl oleanolate	14162-53-9	[34]
15	chikusetsusaponin Iva	51415-02-2	[34]
16	serratagenic acid	6488-64-8	[34]
17	akebonoic acid	104777-60-8	[34]
18	Taxisterone	19536-24-4	[34]
19	7α-hydroxysitosterol	34427-61-7	[34]
20	7β-hydroxysitosterol	15140-59-7	[34]
21	stigmasterol-β-D-glucoside	19716-26-8	[34]
22	4-hydroxy-3-methoxy-benzoic acid	121-34-6	[33]
23	uridine	58-96-8	[32]
24	Inulin	22413-78-1	[35]
25	quercetin-3-O-glucoside	482-35-9	[36]
26	kaempferol-3-O-glucoside	480-10-4	[36]
27	ginsenoside Ro	34367-04-9	[36]
28	chikusetsusaponin IV	7518-22-1	[36]
29	oleanonic acid	17990-42-0	[36]
30	gluconic acid	526-95-4	[36]

**Table 2:** Top 10 ingredients of the “Component-Target-Disease” network diagram.

Molecule Name	Degree	Betweenness Centrality	Closeness Centrality
Flavonols	87	0.020940631	0.39269813
Ginsenoside Ro	86	0.01486894	0.391304348
Chikusetsusaponin IV	84	0.013922591	0.389920424
Oleanonic acid	82	0.010200463	0.389920424
Ecdysterone 20,22-monoacetonide	79	0.01687878	0.387181738
Ajugasterone c	73	0.011092969	0.38381201
Oleanolic acid	71	0.005200213	0.382480486
Serratagenic acid	71	0.005753417	0.382480486
Rubrosterone	67	0.008512648	0.379844961
Pfaffic acid	67	0.004583201	0.379844961

## Data Availability

All relevant data are contained in the article, the original contributions presented in the study are included in the article, and further inquiries can be directed to the corresponding author.

## Supplementary Materials

The following supporting information can be downloaded at: https://www.mdpi.com/article/10.3390/ph18111614/s1, Table S1. The top 50 significantly enriched GO terms in BP, CC, and MF for the shared targets of Pg and reproductive system injury. Table S2. The top 50 significantly enriched KEGG pathways for the shared targets of Pg and reproductive system injury. Table S3. Effects of different BPA doses and exposure durations on sperm morphology in mice. Figure S1. Assessment of sperm morphology in mice after 28-day administration of Pg (400 mg/kg). Figure S2. Original Western blot images.
