# Supplementary material for: Pfaffia glomerata Ameliorates BPA-Induced Reproductive Impairments in Mice by Suppressing Apoptosis via PI3K/AKT Signaling Activation
Source: Pharmaceuticals (Basel). 2025 Oct 25;18(11):1614. doi: 10.3390/ph18111614 (PMC12655148; doi:10.3390/ph18111614)
Supplement: Supplementary file 1 [file pharmaceuticals-18-01614-s001.zip › pharmaceuticals-3910427-supplementary.pdf]

Table S1. The top 50 significantly enriched GO terms in BP, CC, and MF for the shared targets of Pg and reproductive system injury.

ONTOL-OGY	ID	Description	FoldEnric-hment	zScore	pvalue	p.adjust	geneID	Count
BP	GO:0009410	response to xenobiotic stimulus	7.176766	19.59427	4.20E-39	2.09E-35	RORC/CES2/NOS2/HSD11B2/PTGS2/SLC6A4/CYP2C19/TOP1/SLC6A2/SRD5A2/RORA/BCHE/MDM2/SLC6A3/UGT2B7/EGFR/NTRK1/MAOB/CYP2C9/ABCB11/KCNH2/TYMS/PDE4B/CCNT1/REN/CA9/ADORA2A/ABCB1/CYP1B1/MMP2/CDK1/AKR1C1/LCK/ABCC1/SLC22A12/AHR/CYP1A2/CBR1/CYP1A1/SRC/TNF/DRD2/PDE4A/ADAM17/NOS1/SRD5A1/CASP3/SLC10A1/ATP1A1/PCNA/DRD1/DRD3/NR1I2/RET/HSF1/HTR2B/CYP2D6/HTR1B/CD38/ADA/TPMT/FBP1/HSP90AA1/CYP3A4/GRIN1/CHUK/HDAC5/PDE3A/NFE2L2	69
BP	GO:0008202	steroid metabolic process	7.299113	17.14763	7.46E-30	1.86E-26	RORC/HSD11B1/HSD11B2/CYP19A1/PPARD/SERPINA6/G6PD/CYP51A1/NPC1L1/CYP17A1/NR3C1/CYP2C19/HMGCR/NR1H4/ESR1/SRD5A2/RORA/CES1/FDFT1/ACLY/UGT2B7/AKR1B1/CYP2C9/ABCB11/PDE8B/CETP/CYP1B1/AKR1C2/AKR1C1/AKR1C4/AKR1C3/CYP1A2/CBR1/CYP1A1/SREBF2/TNF/SQLE/APP/SRD5A1/HSD17B3/ATP1A1/NR1I2/STS/DHCR7/GBA2/CYP2D6/NFKB1/HSD17B10/CYP3A4/PDGFRA/CFTR/CACNA1H	52
BP	GO:0097305	response to alcohol	8.202018	17.36254	9.36E-29	1.55E-25	POLB/PPARA/G6PD/PTGER2/PTGER4/CES1/MDM2/SLC6A3/PTGFR/SMO/MAOB/ABCB11/AKT1/AVPR1A/PARP1/TGFBR1/TYMS/ST6GAL1/CYP1B1/CDK1/AKR1C2/AHR/AKR1C3/FYN/TNF/PTGDR/DRD2/CNR1/HPGD/OPRM1/OPRK1/BCL2L1/GLRA1/GLRA2/SLC10A1/HDAC6/IL2/HTR1B/CDA/P2RY4/DRD4/FBP1/BLM/GRIN1/ACACA/CFTR	46
BP	GO:0030522	intracellular receptor signaling pathway	6.633033	16.25738	2.59E-28	3.22E-25	RORC/AR/PPARG/PPARA/PPARD/FABP5/ESR2/NR1H3/NR3C1/TLR9/NR1H4/PGR/NR3C2/ESR1/RORA/NR1I3/MAPK8/MAPK1/SIRT2/HDAC1/AKT1/PARP1/NR4A2/BTK/PIM1/ALOX15/ESRRA/PTPRS/AURKB/PKN1/PRKDC/AHR/AKR1C3/IKBKB/SRC/TNF/VDR/NR1H2/JAK2/STAT3/HDAC6/NR1I2/THRB/	

BP	GO:0062197	cellular response to chemical stress	7.127603	16.21015	4.02E-27	4.00E-24	PPP2CA/KDM3A/KDM4C/TRIM24/KDM1A/TLR4/NR4A1/RORB/TLR8/PRCP FABP1/PTGS2/G6PD/MAPK3/ALOX5/MDM2/MTOR/PIK3CA/MAPK8/MAPK1/EGFR/MET/MAPK9/SIRT2/MMP9/AKR1B1/AKT1/PARP1/NR4A2/BTK/CYP1B1/MMP2/CDK1/MYLK/MAPT/ABCC1/FYN/SRC/MMP3/IL6/NOS3/EIF2AK3/CASP3/JUN/HDAC6/PCNA/LRRK2/DHFR/HSF1/KDM6B/FBP1/SCN2A/PDGFRA/CHUK/NFE2L2/SPHK1/NOX1/KEAP1	48
BP	GO:0048511	rhythmic process	7.152602	15.89972	4.36E-26	3.61E-23	RORC/NOS2/PPARG/PPARA/NR1H3/SLC6A4/TOP1/PGR/ESR1/RORA/KDM5C/MTOR/MAPK8/EGFR/MAPK9/NTRK1/HDAC1/PARP1/TYMS/MAPK10/HCRTR2/CA12/ADORA1/ADORA2A/CYP1B1/MMP2/GSK3B/CDK1/TOP2A/PRKDC/AHR/NTRK2/SRC/TNF/DRD2/NOS3/CREBBP/OPRK1/CASP3/PPP1CC/PCNA/DRD3/OGT/DRD4/RORB/PDGFRA	46
BP	GO:0002237	response to molecule of bacterial origin	6.007275	14.4514	9.11E-24	6.38E-21	NOS2/PTGS2/PTGER2/MAPK3/NR1H3/TLR9/NR1H4/PTGER4/PTGIR/PTGFR/MAPK8/MAPK1/MAPK14/MMP9/MAOB/AKT1/BTK/PDE4B/REN/AHR/CYP1A1/SRC/TNF/PTGER1/CNR1/MMP3/IL6/ADAM17/NOS1/NOS3/JAK2/HPGD/OPRK1/PTAFR/PRKCA/CASP3/MMP8/IDO1/CNR2/HSF1/RPS6KA3/SERPINE1/NFKB1/TLR4/C5AR1/NR4A1/CHUK/HDAC5	48
BP	GO:0050878	regulation of body fluid levels	5.991039	14.42443	1.03E-23	6.38E-21	PTPN6/HSD11B2/NR1H3/SLC6A4/SLC6A3/PIK3CA/EGFR/MAPK14/PIK3CB/SYK/AKR1B1/PIK3CG/F10/F9/ADORA1/ADORA2A/XDH/ALOX12/AVPR2/PLA2G4A/LCK/F2/SRC/VDR/DRD2/NR1H2/IL6/NOS3/JAK2/OPRK1/P2RY12/PRKCA/CHRM3/CHRM1/ADRA2A/ADRA2B/ADA/P2RY2/SERPINE1/TUBB1/TLR4/TFPI/PDGFRA/PLAU/F13A1/NFE2L2/CFTR/FAP	48
BP	GO:0032496	response to lipopolysaccharide	6.104376	14.2982	4.20E-23	2.32E-20	NOS2/PTGS2/PTGER2/MAPK3/NR1H3/TLR9/NR1H4/PTGER4/PTGIR/PTGFR/MAPK8/MAPK1/MAPK14/MMP9/MAOB/AKT1/BTK/PDE4B/REN/CYP1A1/SRC/TNF/PTGER1/CNR1/MMP3/IL6/ADAM17/NOS1/NOS3/JAK2/HPGD/OPRK1/PTAFR/PRKCA/CASP3/MMP8/IDO1/CNR2/HSF1/RPS6KA3/SERPINE1/NFKB1/TLR4/NR4A1/CHUK/HDAC5	46

BP	GO:0070482	response to oxygen levels	6.000912	14.13091	8.69E-23	4.00E-20	POLB/NOS2/FABP1/PPARG/HSD11B2/TERT/PTGS2/PPARA/PPARD/SLC6A4/RORA/MDM2/CHRNA7/EGLN1/MTOR/PGK1/PIK3CB/SIRT2/AKT1/P2RX3/NR4A2/DPP4/CA9/ADORA1/MMP2/CYP1A1/SRC/TNF/DRD2/ADAM17/NOS1/CREBBP/OPRD1/PSEN2/CASP3/MMP14/MDM4/HSF1/CD38/ADA/SCN2A/PLAU/SLC9A1/PIN1/NFE2L2/NOX1	46
BP	GO:0048545	response to steroid hormone	6.148349	14.20883	9.53E-23	4.00E-20	AR/HSD11B2/PTGS2/PPARA/PPARD/ESR2/PTGER2/NR1H3/NR3C1/PGR/NR3C2/ESR1/SRD5A2/BCHE/MDM2/PIK3CA/MAPK1/EGFR/MAOB/HDAC1/AVPR1A/PARP1/TYMS/FLT3/ESRRA/CYP1B1/PKN1/AKR1C3/SRC/TNF/IL6/JAK2/SRD5A1/PTAFR/CASP3/HDAC6/ATP1A1/PCNA/HTR1B/CD38/KDM3A/KDM4C/KDM1A/TFPI/PDE3A	45
BP	GO:0050727	regulation of inflammatory response	5.43305	13.7515	9.64E-23	4.00E-20	CD81/PTGES/PTPN6/PPARG/PTGS2/CYP19A1/FABP4/PPARA/PPARD/NR1H3/TLR9/NR1H4/ALOX5/PTGER4/ESR1/RORA/MAPK14/MMP9/SYK/AGTR1/PIK3CG/BTK/ADORA1/ADORA2A/ALOX15/ABCC1/AHR/FYN/PLA2G2A/SRC/TNF/PTGER3/APP/CNR1/MMP3/IL6/BRD4/JAK2/MMP8/STAT3/IL2/IDO1/LRRK2/ADA/SERPINE1/NFKB1/TLR4/FPR2/SPHK1/NT5E	50
BP	GO:0036293	response to decreased oxygen levels	6.213948	14.14959	1.90E-22	7.27E-20	NOS2/FABP1/PPARG/HSD11B2/TERT/PTGS2/PPARA/PPARD/SLC6A4/RORA/MDM2/CHRNA7/EGLN1/MTOR/PGK1/PIK3CB/SIRT2/AKT1/P2RX3/NR4A2/DPP4/CA9/ADORA1/MMP2/CYP1A1/SRC/TNF/DRD2/ADAM17/NOS1/CREBBP/OPRD1/PSEN2/CASP3/MMP14/MDM4/HSF1/CD38/ADA/SCN2A/PLAU/SLC9A1/PIN1/NFE2L2	44
BP	GO:0071466	cellular response to xenobiotic stimulus	8.331611	15.27049	2.50E-22	8.50E-20	RORC/CES2/NOS2/CYP2C19/RORA/BCHE/UGT2B7/EGFR/CYP2C9/ABCB11/KCNH2/PDE4B/REN/ABCB1/CYP1B1/AKR1C1/ABCC1/AHR/CYP1A2/CBR1/CYP1A1/PDE4A/NOS1/SLC10A1/PCNA/NR1I2/HSF1/CYP2D6/HTR1B/ADA/TPMT/FBP1/CYP3A4/GRIN1/NFE2L2	35
BP	GO:0001666	response to hypoxia	6.344345	14.18424	2.56E-22	8.50E-20	NOS2/FABP1/PPARG/HSD11B2/TERT/PTGS2/PPARA/PPARD/SLC6A4/RORA/MDM2/CHRNA7/EGLN1/MTOR/PGK1/PIK3CB/SIRT2/P2RX3/NR4A2/DPP4/	43

							CA9/ADORA1/MMP2/CYP1A1/SRC/TNF/DRD2/ADAM17/NOS1/CREBBP/OPRD1/PSEN2/CASP3/MMP14/MDM4/HSF1/CD38/ADA/SCN2A/PLAU/SLC9A1/PIN1/NFE2L2	
BP	GO:0043410	positive regulation of MAPK cascade	5.055596	13.31813	3.54E-22	1.10E-19	PTPN1/PLA2G1B/CD81/AR/PTPN11/MAPK3/GPBAR1/TLR9/CHRNA7/EGFR/SYK/NTRK1/FLT1/MAP2K1/CCR1/PIK3CG/HCRTR1/CSF1R/PDE8B/ADORA1/ALOX15/FLT3/XDH/KDR/NOX4/NTRK2/PLA2G2A/SRC/TNF/DRD2/APP/IL6/JAK2/OPRM1/OPRK1/PRKCA/LRRK2/RET/HTR2B/ADRA2A/ADRA2B/HTR2C/DRD4/GPR55/TLR4/NTRK3/FPR2/C5AR1/PDGFRA/SPHK1/PDGFRB/NOX1	52
BP	GO:0007200	phospholipase C-activating G protein-coupled receptor signaling pathway	12.22436	16.91019	3.93E-22	1.15E-19	LTB4R/CHRM2/ESR1/S1PR1/AGTR1/HCRTR2/F2/PTGER3/DRD2/OPRM1/OPRD1/OPRK1/PTAFR/P2RY12/DRD1/DRD3/CHRM3/CHRM1/HTR2B/ADRA2A/HTR2C/P2RY4/P2RY2/FPR2/C5AR1/FPR1/NTSR2	27
BP	GO:1901653	cellular response to peptide	5.649795	13.54893	1.10E-21	3.03E-19	PTPN1/PLA2G1B/PPARG/PTPN11/MAPK3/NR1H4/MDM2/MTOR/PIK3CA/MAPK1/GRB2/AGTR1/AGTR2/CA2/AKT2/AKT1/PARP1/NR4A2/CYP1B1/GSK3B/PRKDC/ABCC1/SLC22A12/FYN/SRC/PTK2/TNF/APP/PSEN1/JAK1/JAK2/SRD5A1/PRKCA/STAT3/HSF1/OGT/FBP1/NFKB1/CTSD/TLR4/PIK3R1/FPR2/NR4A1/HDAC5/SLC9A1/NFE2L2	46
BP	GO:0034614	cellular response to reactive oxygen species	9.480853	15.56617	1.20E-21	3.13E-19	FABP1/MAPK3/MDM2/MAPK8/MAPK1/EGFR/MET/MAPK9/MMP9/AKT1/BTK/CYP1B1/MMP2/CDK1/MAPT/FYN/SRC/MMP3/IL6/NOS3/JUN/HDAC6/PCNA/LRRK2/DHFR/HSF1/KDM6B/PDGFRA/CHUK/NFE2L2/SPHK1	31
BP	GO:0034599	cellular response to oxidative stress	7.07611	14.32955	1.57E-21	3.90E-19	FABP1/G6PD/MAPK3/ALOX5/MDM2/MAPK8/MAPK1/EGFR/MET/MAPK9/SIRT2/MMP9/AKT1/PARP1/NR4A2/BTK/CYP1B1/MMP2/CDK1/MAPT/ABCC1/FYN/SRC/MMP3/IL6/NOS3/JUN/HDAC6/PCNA/LRRK2/DHFR/HSF1/KDM6B/PDGFRA/CHUK/NFE2L2/SPHK1/KEAP1	38
BP	GO:00	response to oxidative	5.453527	13.35922	1.73E-21	4.09E-19	FABP1/PTGS2/G6PD/PTGS1/MAPK3/ALOX5/MDM2/MAPK8/MAPK1/EGFR/	47

	06979	stress					MET/MAPK9/SIRT2/MMP9/ABCB11/AKT1/PARP1/NR4A2/BTK/CYP1B1/MMP2/CDK1/MAPT/ABCC1/FYN/SRC/APP/MMP3/PSEN1/IL6/NOS3/JAK2/CASP3/JUN/HDAC6/MMP14/PCNA/LRRK2/DHFR/HSF1/CD38/KDM6B/PDGFRA/CHUK/NFE2L2/SPHK1/KEAP1	
BP	GO:0006690	icosanoid metabolic process	10.34453	15.59427	9.33E-21	2.11E-18	PLA2G1B/PTGES/CES2/PTGS2/PTGS1/FABP5/FAAH/CYP2C19/ALOX5/SYK/AKR1B1/CYP2C9/AVPR1A/ALOX15/ALOX12/CYP1B1/AKR1C2/AKR1C1/AKR1C4/PLA2G4A/ABCC1/AKR1C3/CYP1A2/CBR1/CYP1A1/HPGD/CYP2D6/LTA4H	28
BP	GO:0043434	response to peptide hormone	5.060924	12.79286	1.53E-20	3.30E-18	PTPN1/PLA2G1B/PPARG/HSD11B2/PTPN11/PTGS2/PPARA/MAPK3/NR1H4/SRD5A2/MDM2/MTOR/PIK3CA/MAPK1/MAPK14/GRB2/AGTR1/AGTR2/CA2/AKT2/AKT1/PARP1/NR4A2/CYP1B1/GSK3B/PRKDC/SLC22A12/SRC/PTK2/JAK1/JAK2/SRD5A1/OPRK1/PRKCA/STAT3/HSF1/OGT/FBP1/NFKB1/TRIM24/CTSD/PIK3R1/NR4A1/CHUK/HDAC5/SLC9A1/NFE2L2/LTA4H	48
BP	GO:0007188	adenylate cyclase-modulating G protein-coupled receptor signaling pathway	6.862226	13.85335	1.60E-20	3.33E-18	PTGER2/PTGER4/CHRM2/PTGIR/PTGFR/S1PR1/PDE4B/S1PR3/ADORA1/ADORA2A/AVPR2/PTGER3/PTGER1/DRD2/PDE4A/CNR1/PTGDR2/NOS1/OPRM1/OPRD1/OPRK1/P2RY12/PRKCA/DRD1/DRD3/CHRM3/CHRM1/S1PR2/CNR2/ADRA2A/ADRA2B/HTR1B/DRD4/GPR119/FPR2/FPR1/GRK5	37
BP	GO:0072593	reactive oxygen species metabolic process	6.985351	13.82428	2.94E-20	5.84E-18	NOS2/PPARA/G6PD/ALOX5/EGFR/MAPK14/GRB2/SIRT2/SYK/AGTR1/MAOB/XDH/ALOX12/CYP1B1/MAPT/AKR1C1/NOX4/F2/AKR1C3/FYN/CYP1A2/CBR1/CYP1A1/TNF/MMP3/NOS3/HDAC6/LRRK2/DHFR/TLR4/FPR2/GRIN1/PRCP/NFE2L2/PDGFRB/NOX1	36
BP	GO:0042060	wound healing	5.047683	12.63142	4.39E-20	8.41E-18	PTPN6/PPARG/PPARA/PPARD/SLC6A4/ALOX5/MTOR/PIK3CA/MAPK14/PIK3CB/SYK/TGFBR1/PIK3CG/F10/F9/ADORA2A/ALOX15/KDR/ALOX12/MYLK/PLA2G4A/LCK/F2/SRC/PTK2/TNF/ITGAV/IL6/ADAM17/NOS3/JAK2/P2RY12/PRKCA/ITGB1/MMP12/ADRA2A/ADRA2B/SERPINE1/TUBB1/TLR4/TFPI/PDGFRA/PLAU/PRCP/F13A1/NFE2L2/FAP	47

BP	GO:0042180	cellular ketone metabolic process	7.120408	13.80161	5.29E-20	9.75E-18	AKR1B10/FABP1/PPARG/HSD11B2/PTGS2/CYP19A1/PPARA/PPARD/FABP5/CYP17A1/NR1H3/NR1H4/SRD5A2/CES1/SIRT2/AKR1B1/ABCB11/AKT2/AKT1/AVPR1A/GLO1/AKR1C2/AKR1C1/AKR1C4/AKR1A1/AKR1C3/CBR1/NR1H2/CNR1/SRD5A1/HSD17B3/IDO1/NQO2/HSD17B10/CACNA1H	35
BP	GO:1901654	response to ketone	7.235722	13.74475	1.09E-19	1.94E-17	AR/PTGER2/NR1H3/NR3C1/PTGER4/SRD5A2/PTGFR/PIK3CA/EGFR/MAOB/AKT1/AVPR1A/PARP1/TYMS/CA9/CYP1B1/AKR1C2/AHR/AKR1C3/SRC/PTGDR/JAK2/SRD5A1/BCL2L1/PTAFR/HDAC6/PCNA/HSF1/CD38/CDA/P2RY4/FBP1/ACACA/CFTR	34
BP	GO:0007623	circadian rhythm	7.434003	13.77967	1.63E-19	2.79E-17	RORC/NOS2/PPARG/PPARA/NR1H3/SLC6A4/TOP1/RORA/MTOR/MAPK8/EGFR/MAPK9/NTRK1/HDAC1/PARP1/TYMS/MAPK10/HCRTR2/ADORA1/ADORA2A/GSK3B/CDK1/TOP2A/PRKDC/AHR/NTRK2/TNF/DRD2/PPP1CC/DRD3/OGT/DRD4/RORB	33
BP	GO:0042445	hormone metabolic process	6.734719	13.30165	3.37E-19	5.41E-17	AKR1B10/PTPN11/CYP19A1/CYP17A1/NR3C1/ESR1/SRD5A2/BCHE/UGT2B7/MME/AKR1B1/CYP2C9/PDE8B/REN/DPP4/CYP1B1/AKR1C2/AKR1C1/AKR1C4/AKR1C3/CYP1A2/CYP1A1/SRD5A1/HSD17B3/ATP1A1/DHCR7/CTSL/CTSB/CYP2D6/NFKB1/HSD17B10/ADAM10/CYP3A4/PDGFRA/CACNA1H	35
BP	GO:0051347	positive regulation of transferase activity	5.28133	12.47527	3.40E-19	5.41E-17	PTPN1/PLA2G1B/MAPK3/CHRNA7/MAPK1/ALK/EGFR/MET/AGTR1/NTRK1/FLT1/MAP2K1/AKT1/PIK3CG/CSF1R/FLT3/KDR/AURKB/MAPT/CALM1/NOX4/F2/NTRK2/SRC/PTK2/TNF/CCND3/ADAM17/JAK2/PCNA/LRRK2/RET/HTR2B/ADRA2A/ADRA2B/PPP2CA/DRD4/TLR4/NTRK3/HSP90AB1/HSP90AA1/PDGFRA/PDGFRB	43
BP	GO:0046942	carboxylic acid transport	5.417874	12.55513	3.48E-19	5.41E-17	PLA2G1B/PTGES/NOS2/FABP1/PPARG/PTGS2/FABP4/PPARA/PPARD/FABP5/SLC6A4/SLC6A2/CES1/SLC6A3/SLC22A6/FABP2/SYK/ABCB11/AKT2/AKT1/AVPR1A/ABCG2/ADORA1/ADORA2A/ABCB1/AKR1C1/AKR1C4/PLA2G4A/ABCC1/NTRK2/PLA2G2A/TNF/DRD2/PSEN1/NTSR1/ITGB1/SLC10A1/DRD3/SLC16A1/HTR1B/DRD4/SLC6A5	42

BP	GO:0015849	organic acid transport	5.402783	12.53004	3.86E-19	5.82E-17	PLA2G1B/PTGES/NOS2/FABP1/PPARG/PTGS2/FABP4/PPARA/PPARD/FABP5/SLC6A4/SLC6A2/CES1/SLC6A3/SLC22A6/FABP2/SYK/ABCB11/AKT2/AKT1/AVPR1A/ABCG2/ADORA1/ADORA2A/ABCB1/AKR1C1/AKR1C4/PLA2G4A/ABCC1/NTRK2/PLA2G2A/TNF/DRD2/PSEN1/NTSR1/ITGB1/SLC10A1/DRD3/SLC16A1/HTR1B/DRD4/SLC6A5	42
BP	GO:0006631	fatty acid metabolic process	5.067234	12.24735	6.41E-19	9.38E-17	PLA2G1B/PTGES/CES2/FABP1/PPARG/SCD/PTGS2/PPARA/PPARD/PTGS1/FABP5/FAAH/NR1H3/CYP2C19/ALOX5/CES1/ACLY/FABP2/MAPK14/SIRT2/AKR1B1/CYP2C9/ABCB11/AKT2/AKT1/AVPR1A/ALOX15/ALOX12/CYP1B1/AKR1C2/AKR1C1/AKR1C4/PLA2G4A/AKR1C3/CYP1A2/CBR1/CYP1A1/NR1H2/CNR1/HPGD/CYP2D6/HSD17B10/CYP3A4/ACACA	44
BP	GO:0071375	cellular response to peptide hormone stimulus	5.791178	12.67473	6.66E-19	9.46E-17	PTPN1/PLA2G1B/PPARG/PTPN11/MAPK3/NR1H4/MDM2/MTOR/PIK3CA/MAPK1/GRB2/AGTR1/AGTR2/CA2/AKT2/AKT1/PARP1/NR4A2/CYP1B1/GSK3B/PRKDC/SLC22A12/SRC/PTK2/JAK1/JAK2/SRD5A1/PRKCA/STAT3/HSF1/OGT/FBP1/NFKB1/CTSD/PIK3R1/NR4A1/HDAC5/SLC9A1/NFE2L2	39
BP	GO:0000302	response to reactive oxygen species	7.279752	13.38283	1.12E-18	1.55E-16	FABP1/MAPK3/MDM2/MAPK8/MAPK1/EGFR/MET/MAPK9/MMP9/AKT1/BTK/CYP1B1/MMP2/CDK1/MAPT/FYN/SRC/MMP3/IL6/NOS3/CASP3/JUN/HDAC6/PCNA/LRRK2/DHFR/HSF1/KDM6B/PDGFRA/CHUK/NFE2L2/SPHK1	32
BP	GO:0043491	phosphatidylinositol 3-kinase/protein kinase B signal transduction	5.869148	12.62596	1.20E-18	1.56E-16	PTPN6/PPARA/PPARD/MTOR/PIK3CA/EGFR/PIK3CB/NTRK1/FLT1/AKT1/TGFBR1/PIK3CD/PIK3CG/CSF1R/FLT3/XDH/KDR/AKR1C2/NOX4/F2/AKR1C3/NTRK2/SRC/PTK2/TNF/DRD2/JAK2/P2RY12/ITGB1/STAT3/DRD3/RET/HTR2B/PPP2CA/NTRK3/PIK3R1/PDGFRA/PDGFRB	38
BP	GO:0071214	cellular response to abiotic stimulus	5.53065	12.42851	1.22E-18	1.56E-16	PTGS2/CDC25A/MAPK3/PTGER4/MDM2/MTOR/PIK3CA/MAPK8/MME/EGFR/MAPK14/GRB2/MMP9/AKR1B1/AKT2/AVPR1A/PARP1/CHEK1/MAP3K14/MMP2/AURKB/MYLK/MMP3/CREBBP/BCL2L1/PTAFR/CASP3/MMP1/PCNA/HSF1/FBP1/BLM/NFKB1/KDM1A/TLR4/SCN2A/PIK3R1/SLC9A1/TLR8/NOX1	40

BP	GO:0104004	cellular response to environmental stimulus	5.53065	12.42851	1.22E-18	1.56E-16	PTGS2/CDC25A/MAPK3/PTGER4/MDM2/MTOR/PIK3CA/MAPK8/MME/EGFR/MAPK14/GRB2/MMP9/AKR1B1/AKT2/AVPR1A/PARP1/CHEK1/MAP3K14/MMP2/AURKB/MYLK/MMP3/CREBBP/BCL2L1/PTAFR/CASP3/MMP1/PCNA/HSF1/FBP1/BLM/NFKB1/KDM1A/TLR4/SCN2A/PIK3R1/SLC9A1/TLR8/NOX1	40
BP	GO:0120254	olefinic compound metabolic process	8.266956	13.81947	1.35E-18	1.68E-16	AKR1B10/HSD11B2/PTGS2/CYP19A1/PTGS1/FAAH/CYP17A1/CYP2C19/ALOX5/SRD5A2/AKR1B1/CYP2C9/ALOX15/ALOX12/CYP1B1/AKR1C2/AKR1C1/AKR1C4/PLA2G4A/AKR1C3/CYP1A2/CBR1/CYP1A1/SRD5A1/HSD17B3/CYP2D6/CYP3A4/GRIN1/CACNA1H	29
BP	GO:2000377	regulation of reactive oxygen species metabolic process	8.678295	13.99873	1.40E-18	1.69E-16	PPARA/G6PD/ALOX5/EGFR/MAPK14/GRB2/SIRT2/SYK/AGTR1/XDH/CYP1B1/MAPT/AKR1C1/F2/AKR1C3/FYN/CBR1/TNF/MMP3/HDAC6/LRRK2/DHFR/TLR4/FPR2/GRIN1/PRCP/NFE2L2/PDGFRB	28
BP	GO:0043269	regulation of monoatomic ion transport	4.618093	11.81711	1.67E-18	1.98E-16	PLA2G1B/PDE4D/PTPN6/PTGS2/G6PD/SLC6A4/TLR9/MMP9/MAOB/CA2/KCNH2/AKT1/P2RX3/CCR1/PIK3CG/PDE4B/CA7/ADORA1/ADORA2A/ABCB1/MYLK/CALM1/F2/FYN/DRD2/CNR1/NTSR1/NOS1/NOS3/OPRD1/OPRK1/PSEN2/P2RY12/ITGB1/ATP1A1/DRD1/DRD3/KCNJ1/CHRM1/ADRA2A/HTR1B/DRD4/GRIN1/SLC9A1/TACR2/PDGFRB/CFTR	47
BP	GO:0033559	unsaturated fatty acid metabolic process	10.03933	14.46513	2.51E-18	2.91E-16	PTGES/CES2/SCD/PTGS2/PTGS1/FABP5/FAAH/CYP2C19/ALOX5/AKR1B1/CYP2C9/AVPR1A/ALOX15/ALOX12/CYP1B1/AKR1C2/AKR1C1/AKR1C4/PLA2G4A/AKR1C3/CYP1A2/CBR1/CYP1A1/HPGD/CYP2D6	25
BP	GO:0032102	negative regulation of response to external stimulus	4.658602	11.76563	2.81E-18	3.18E-16	PTPN6/PPARG/CYP19A1/PPARA/PPARD/NR1H3/NR1H4/ALOX5/PTGER4/RORA/SLC6A3/MAPK14/SYK/PARP1/DPP4/ADORA1/ADORA2A/ST6GAL1/ALOX12/ARG1/PTPRS/AURKB/F2/AHR/FYN/SRC/TNF/DRD2/NR1H2/NOS3/MMP12/STAT3/HDAC6/IL2/DRD1/DRD3/ADA/PPM1B/SERPINE1/NFKB1/TFPI/FPR2/PDGFRA/PLAU/NT5E/FAP	46
BP	GO:00	regulation of small	5.441258	12.12746	5.99E-18	6.62E-16	RORC/NOS2/FABP1/PPARG/PTGS2/PPARA/PPARD/FABP5/NR1H3/NR3C1/	39

	62012	molecule metabolic process					NR1H4/RORA/CES1/MTOR/SIRT2/ABCB11/AKT2/AKT1/AVPR1A/PARP1/AKR1C3/SRC/SREBF2/TNF/NR1H2/APP/CNR1/PSEN1/NTSR1/NOS1/NOS3/PTAFR/STAT3/CDA/OGT/PPP2CA/FBP1/NFKB1/WDR5	
BP	GO:0006694	steroid biosynthetic process	7.488799	13.19467	6.15E-18	6.65E-16	CYP19A1/G6PD/CYP51A1/NPC1L1/CYP17A1/NR3C1/HMGCR/NR1H4/SRD5A2/CES1/FDFT1/ACLY/AKR1B1/ABCB11/PDE8B/AKR1C4/AKR1C3/CYP1A1/SREBF2/TNF/SQLE/SRD5A1/HSD17B3/ATP1A1/DHCR7/NFKB1/HSD17B10/CYP3A4/CFTR/CACNA1H	30
BP	GO:0070371	ERK1 and ERK2 cascade	5.424868	12.10127	6.65E-18	7.04E-16	PTPN1/PTPN6/PTPN11/MAPK3/GPBAR1/HMGCR/TLR9/PTGER4/CHRNA7/MAPK1/EGFR/SYK/NTRK1/MAP2K1/CCR1/HCRTR1/CSF1R/PDE8B/ALOX15/KDR/CDK1/NOX4/PLA2G2A/SRC/TNF/DRD2/APP/ITGAV/OPRM1/PRKCA/HTR2B/HTR2C/GPR55/TLR4/FPR2/C5AR1/PDGFRA/PIN1/PDGFRB	39
BP	GO:0018105	peptidyl-serine phosphorylation	5.895438	12.32235	8.78E-18	9.02E-16	PDE4D/PRKCH/PTGS2/TOP1/MTOR/MAPK8/CDK2/AURKA/MAPK1/EGFR/MAPK14/MAPK9/MKNK2/AKT2/AKT1/AKT3/TGFBR1/GSK3B/CDK1/PKN1/PRKDC/IKBKB/NTRK2/TNF/APP/IL6/NOS1/EIF2AK3/OPRD1/PRKCA/HDAC6/LRRK2/RET/RPS6KA3/NTRK3/CHUK	36
BP	GO:0031667	response to nutrient levels	4.429599	11.44353	8.89E-18	9.02E-16	FABP1/PPARG/HSD11B2/PTGS2/PPARA/PPARD/G6PD/MAPK3/SLC6A4/NR1H4/SRD5A2/BCHE/MDM2/MTOR/MAPK8/MAPK1/EGFR/MAPK14/UPP1/SIRT2/NTRK1/AKT1/TYMS/PIM1/TYR/CYP1B1/AKR1C3/CYP1A1/SRC/SREBF2/TNF/VDR/CNR1/GLUL/CREBBP/EIF2AK3/SRD5A1/SLC10A1/SLC16A1/LRRK2/HSF1/HTR2C/OGT/KDM4A/FBP1/TRIM24/NFE2L2	47
BP	GO:0015711	organic anion transport	4.607853	11.53708	1.03E-17	1.02E-15	PLA2G1B/PTGES/NOS2/FABP1/PPARG/PTGS2/FABP4/PPARA/PPARD/FABP5/SLC6A4/SLC6A2/CES1/SLC6A3/SLC22A6/FABP2/SYK/ABCB11/AKT2/AKT1/AVPR1A/CA4/ABCG2/ADORA1/ADORA2A/ABCB1/AKR1C1/AKR1C4/PLA2G4A/ABCC1/SLC22A12/NTRK2/PLA2G2A/TNF/DRD2/PSEN1/NTSR1/ITGB1/SLC10A1/DRD3/SLC16A1/HTR1B/DRD4/SLC6A5/CFTR	45
CC	GO:00	membrane raft	6.76977523	14.2747463	7.85E-22	1.93E-19	PTGS2/MAPK3/SLC6A4/SLC6A3/CHRNA7/SLC22A6/MAPK1/MME/EGFR/P	40

	45121		7	5			GK1/S1PR1/SMO/TGFBR1/BTK/DPP4/ABCG2/KDR/MAPT/LCK/IKBKB/FYN/SRC/TNF/APP/TRPM8/CNR1/PSEN1/NTSR1/ADAM17/NOS1/NOS3/JAK2/ITGB1/HDAC6/ATP1A1/LRRK2/PPP2CA/CTSD/TFPI/SLC9A1	
CC	GO:0098857	membrane microdomain	6.74618717	14.24158345	8.96E-22	1.93E-19	PTGS2/MAPK3/SLC6A4/SLC6A3/CHRNA7/SLC22A6/MAPK1/MME/EGFR/PGK1/S1PR1/SMO/TGFBR1/BTK/DPP4/ABCG2/KDR/MAPT/LCK/IKBKB/FYN/SRC/TNF/APP/TRPM8/CNR1/PSEN1/NTSR1/ADAM17/NOS1/NOS3/JAK2/ITGB1/HDAC6/ATP1A1/LRRK2/PPP2CA/CTSD/TFPI/SLC9A1	40
CC	GO:0042734	presynaptic membrane	6.666404417	10.6801762	6.59E-13	9.49E-11	ADORA3/SLC6A4/CHRM2/SLC6A2/GRIK2/SLC6A3/GABRA5/ADORA1/ADORA2A/PTPRS/DRD2/CNR1/PSEN1/OPRD1/OPRK1/PSEN2/DRD1/CHRM1/ADRA2A/HTR1B/P2RY4/SLC6A5/SCN2A	23
CC	GO:0043025	neuronal cell body	3.769155598	8.995646771	2.42E-12	2.61E-10	PTPRF/CYP17A1/TOP1/CHRM2/SLC6A2/GRIK2/SRD5A2/SLC6A3/AURKA/MME/SIRT2/GABRA5/NTRK1/MAOB/HDAC1/ADORA1/ADORA2A/PTPRS/MAPT/FYN/SRC/TNF/DRD2/APP/NTSR1/SRD5A1/OPRM1/OPRK1/GLRA1/CASP3/HDAC6/LRRK2/CNR2/RET/ADRA2A/HSP90AB1/HSP90AA1/GRIA2	38
CC	GO:0097060	synaptic membrane	3.901573235	8.481800066	5.81E-11	5.02E-09	SIGMAR1/ADORA3/SLC6A4/CHRM2/SLC6A2/GRIK2/SLC6A3/CHRNA7/GABRA5/ADORA1/ADORA2A/PTPRS/SRC/DRD2/CNR1/PSEN1/OPRD1/OPRK1/GLRA2/PSEN2/ITGB1/DRD1/CHRM3/CHRM1/ADRA2A/HTR1B/P2RY4/SLC6A5/SCN2A/ADAM10/GRIA2/GRIN1	32
CC	GO:0045177	apical part of cell	3.413095015	7.672710178	9.33E-10	6.71E-08	PDE4D/FABP1/NPC1L1/TLR9/FABP2/EGFR/CA2/ABCB11/REN/DPP4/CA4/CA12/ABCG2/ABCB1/AKR1A1/ABCC1/SLC22A12/CA14/APP/SLC5A1/ADAM17/SLC5A2/ATP1A1/SLC16A1/CTSL/CTSB/P2RY4/HSP90AA1/C5AR1/SLC9A1/PDGFRB/CFTR/FAP	33
CC	GO:0150034	distal axon	4.27093173	7.889099492	2.62E-09	1.62E-07	SIGMAR1/CHRM2/GRIK2/SLC6A3/SIRT2/ADORA1/PTPRS/MAPT/NTRK2/SRC/DRD2/APP/CNR1/PSEN1/NTSR1/OPRD1/OPRK1/CHRM1/LRRK2/ADRA2A/HTR1B/HSP90AB1/HSP90AA1/GRIN1	24
CC	GO:00	vesicle lumen	3.86043318	7.56328601	4.81E-09	2.60E-07	PTPN6/FABP5/ALOX5/ACLY/MAPK1/EGFR/MAPK14/CFD/ARG1/TTR/APP/	26

	31983			6			MMP8/CDA/ADA/HEXB/FUCA1/GGH/SERPINE1/NFKB1/CTSD/HSP90AB1/HSP90AA1/CSNK2B/F13A1/PSMB1/DPP7	
CC	GO:0045178	basal part of cell	3.967532209	7.585467726	5.50E-09	2.64E-07	CD81/TLR9/SLC22A6/AURKA/EGFR/MET/CA12/CA9/ADORA1/ABCC1/CA14/HPGD/SLC10A1/ATP1A1/SLC16A1/CHRM3/ADRA2A/CD38/P2RY4/HSP90AA1/C5AR1/SLC40A1/SLC9A1/PRCP/FAP	25
CC	GO:0016323	basolateral plasma membrane	4.176022136	7.413735077	1.83E-08	7.72E-07	CD81/TLR9/SLC22A6/AURKA/EGFR/CA12/CA9/ADORA1/ABCC1/CA14/HPGD/SLC10A1/ATP1A1/SLC16A1/CHRM3/ADRA2A/CD38/P2RY4/HSP90AA1/C5AR1/SLC40A1/SLC9A1	22
CC	GO:0060205	cytoplasmic vesicle lumen	3.72337638	7.189656927	1.97E-08	7.72E-07	PTPN6/FABP5/ALOX5/ACLY/MAPK1/MAPK14/CFD/ARG1/TTR/APP/MMP8/CDA/ADA/HEXB/FUCA1/GGH/SERPINE1/NFKB1/CTSD/HSP90AB1/HSP90AA1/CSNK2B/F13A1/PSMB1/DPP7	25
CC	GO:0009925	basal plasma membrane	3.892620761	7.156505401	3.23E-08	1.16E-06	CD81/TLR9/SLC22A6/AURKA/EGFR/MET/CA12/CA9/ADORA1/ABCC1/CA14/HPGD/SLC10A1/ATP1A1/SLC16A1/CHRM3/ADRA2A/CD38/P2RY4/HSP90AA1/C5AR1/SLC40A1/SLC9A1	23
CC	GO:1904813	ficolin-1-rich granule lumen	5.85530963	7.877122069	4.24E-08	1.41E-06	ALOX5/ACLY/MAPK1/MAPK14/MMP9/CFD/CTSB/CDA/CTSD/HSP90AB1/HSP90AA1/CSNK2B/CAPN1/PSMB1/LTA4H	15
CC	GO:0101002	ficolin-1-rich granule	4.709567962	7.362410672	5.93E-08	1.83E-06	ALOX5/ACLY/MAPK1/MAPK14/MMP9/CFD/CTSB/CDA/CTSD/HSP90AB1/FPR2/HSP90AA1/FPR1/CSNK2B/CAPN1/PRCP/PSMB1/LTA4H	18
CC	GO:0044306	neuron projection terminus	5.672331204	7.702333997	6.52E-08	1.86E-06	CHRM2/GRIK2/SLC6A3/MME/ADORA1/NTRK2/DRD2/NTSR1/OPRD1/OPRK1/CHRM1/LRRK2/ADRA2A/HTR1B/GRIN1	15
CC	GO:0034774	secretory granule lumen	3.607743573	6.852015758	6.89E-08	1.86E-06	PTPN6/FABP5/ALOX5/ACLY/MAPK1/MAPK14/CFD/ARG1/TTR/APP/MMP8/CDA/HEXB/FUCA1/GGH/SERPINE1/NFKB1/CTSD/HSP90AB1/HSP90AA1/CSNK2B/F13A1/PSMB1/DPP7	24
CC	GO:0043679	axon terminus	6.050486618	7.784850455	7.91E-08	2.01E-06	CHRM2/GRIK2/SLC6A3/ADORA1/NTRK2/DRD2/NTSR1/OPRD1/OPRK1/CHRM1/LRRK2/ADRA2A/HTR1B/GRIN1	14
CC	GO:00	caveola	7.08349652	8.01750368	1.16E-07	2.78E-06	PTGS2/MAPK3/SLC6A3/SLC22A6/MAPK1/SMO/SRC/NOS3/JAK2/HDAC6/L	12

	05901		8	3			RRK2/TFPI	
CC	GO:0098978	glutamatergic synapse	3.195367016	6.515709614	1.27E-07	2.88E-06	MAPK3/CHRM2/GRIK2/EGLN1/AURKA/MAPK14/AKT1/ADORA2A/PTPRS/GSK3B/SRC/DRD2/CNR1/PSEN1/JAK2/ITGB1/PPP1CC/DRD1/CHRM1/LRRK2/ADRA2A/OGT/P2RY4/DRD4/SCN2A/ADAM10/PIN1	27
CC	GO:0016324	apical plasma membrane	3.130600041	6.268013068	3.23E-07	6.98E-06	PDE4D/NPC1L1/TLR9/EGFR/ABCB11/DPP4/CA4/CA12/ABCG2/ABCB1/AKR1A1/ABCC1/SLC22A12/CA14/SLC5A1/ADAM17/SLC5A2/ATP1A1/SLC16A1/CTSL/CTSB/P2RY4/HSP90AA1/SLC9A1/PDGFRB/CFTR	26
CC	GO:0031253	cell projection membrane	3.281619861	6.291088242	3.96E-07	8.16E-06	EGFR/GABRA5/SMO/AKT2/DPP4/CA4/CA9/ABCG2/ADORA1/ADORA2A/MAPT/SLC22A12/SRC/DRD2/PDE4A/ITGAV/SLC5A1/ADAM17/OPRD1/P2RY12/ITGB1/DRD1/HSP90AA1/FAP	24
CC	GO:0044853	plasma membrane raft	5.568589454	7.073597925	6.12E-07	1.20E-05	PTGS2/MAPK3/SLC6A3/CHRNA7/SLC22A6/MAPK1/SMO/SRC/NOS3/JAK2/HDAC6/LRRK2/TFPI	13
CC	GO:0005925	focal adhesion	2.989314054	5.991990328	7.78E-07	1.46E-05	CD81/MAPK3/SLC6A4/MAPK1/MME/EGFR/FLT1/MAP2K1/DPP4/NOX4/SRC/PTK2/ITGAV/ADAM17/JAK1/JAK2/ITGA2B/ITGB1/PPP1CC/MMP14/ADAM10/PLAU/SLC9A1/CAPN1/PDGFRB/FAP	26
CC	GO:0031904	endosome lumen	10.19029325	8.236039535	8.81E-07	1.54E-05	PTPN1/APP/JAK2/LNPEP/CTSL/CTSB/PRF1/CTSD	8
CC	GO:1902554	serine/threonine protein kinase complex	4.982753685	6.769000541	8.93E-07	1.54E-05	CDK2/CDK9/TGFBR1/CCNB3/CCNT1/CDK6/CDK1/PRKDC/IKBKB/CCNE2/CCND3/PCNA/CHUK/CSNK2B	14
CC	GO:0030055	cell-substrate junction	2.919956419	5.852861557	1.21E-06	2.01E-05	CD81/MAPK3/SLC6A4/MAPK1/MME/EGFR/FLT1/MAP2K1/DPP4/NOX4/SRC/PTK2/ITGAV/ADAM17/JAK1/JAK2/ITGA2B/ITGB1/PPP1CC/MMP14/ADAM10/PLAU/SLC9A1/CAPN1/PDGFRB/FAP	26
CC	GO:0045211	postsynaptic membrane	3.507528474	6.092789673	1.34E-06	2.14E-05	SIGMAR1/SLC6A4/CHRM2/GRIK2/SLC6A3/CHRNA7/GABRA5/ADORA1/ADORA2A/PTPRS/DRD2/OPRD1/OPRK1/GLRA2/DRD1/CHRM3/CHRM1/ADRA2A/GRIA2/GRIN1	20
CC	GO:00	neuron to neuron	3.09247093	5.81913229	1.92E-06	2.89E-05	FABP5/SIGMAR1/CHRM2/GRIK2/EGLN1/AURKA/P2RX3/PDE4B/ADORA1/	23

	98984	synapse	8	2			ADORA2A/PTPRS/NTRK2/FYN/NTSR1/NOS1/OPRD1/ITGB1/ATP1A1/CHRM1/ADRA2A/ADAM10/GRIA2/GRIN1	
CC	GO:0061695	transferase complex, transferring phosphorus-containing groups	3.300265428	5.909130379	1.94E-06	2.89E-05	TERT/PIK3CA/CDK2/CDK9/PIK3CB/TGFBR1/PIK3CD/PIK3CG/CCNB3/CCNT1/CDK6/CDK1/PRKDC/IKBKB/CCNE2/CCND3/PCNA/POLA1/PIK3R1/CHUK/CSNK2B	21
CC	GO:0000307	cyclin-dependent protein kinase holoenzyme complex	7.77919708	7.378472571	2.02E-06	2.90E-05	CDK2/CDK9/CCNB3/CCNT1/CDK6/CDK1/CCNE2/CCND3/PCNA	9
CC	GO:0043197	dendritic spine	4.245955521	6.191519846	2.80E-06	3.90E-05	PDE4B/ADORA1/MAPT/NTRK2/PTK2/DRD2/APP/NTSR1/NOS1/OPRD1/ITGB1/PPP1CC/DRD1/GRIA2/GRIN1	15
CC	GO:1902911	protein kinase complex	4.517696675	6.281011428	2.90E-06	3.91E-05	CDK2/CDK9/TGFBR1/CCNB3/CCNT1/CDK6/CDK1/PRKDC/IKBKB/CCNE2/CCND3/PCNA/CHUK/CSNK2B	14
CC	GO:0043204	perikaryon	4.487778154	6.248472905	3.13E-06	4.10E-05	TOP1/GRIK2/SIRT2/PTPRS/FYN/DRD2/APP/NTSR1/OPRM1/OPRK1/GLRA1/HDAC6/LRRK2/CNR2	14
CC	GO:0044309	neuron spine	4.19686933	6.133757573	3.24E-06	4.11E-05	PDE4B/ADORA1/MAPT/NTRK2/PTK2/DRD2/APP/NTSR1/NOS1/OPRD1/ITGB1/PPP1CC/DRD1/GRIA2/GRIN1	15
CC	GO:0005775	vacuolar lumen	4.125331785	6.048729574	4.00E-06	4.94E-05	FABP5/ACLY/MAPK1/ARG1/TTR/CTSL/CTSB/HEXA/HEXB/FUCA1/GGH/CTSD/HSP90AA1/DPP7/PDGFRB	15
CC	GO:0009897	external side of plasma membrane	2.868378841	5.517480554	4.23E-06	5.08E-05	S1PR1/CCR1/F10/CA4/ABCG2/KDR/ABCB1/CXCR1/F2/TNF/CXCR3/TRPM8/ITGAV/GLRA1/ITGA2B/ITGB1/CTSB/ADA/TLR4/GRIA2/PDGFRA/PLAU/TLR8/NT5E	24
CC	GO:0032279	asymmetric synapse	3.089610188	5.55093877	5.45E-06	6.36E-05	FABP5/SIGMAR1/CHRM2/GRIK2/EGLN1/AURKA/PDE4B/ADORA1/ADORA2A/PTPRS/NTRK2/FYN/NOS1/OPRD1/ITGB1/ATP1A1/CHRM1/ADRA2A/ADAM10/GRIA2/GRIN1	21
CC	GO:00	primary lysosome	4.05968134	5.55421402	2.08E-05	0.00023053	FABP5/ACLY/MAPK1/ARG1/TTR/PSEN1/HEXA/HEXB/FUCA1/GGH/FPR1/P	13

	GO:0005766		4.059681344	5.554214028			FABP5/ACLY/MAPK1/ARG1/TTR/PSEN1/HEXA/HEXB/FUCA1/GGH/FPR1/PRCP/DPP7	
CC	GO:0042582	azurophil granule	4.059681344	5.554214028	2.08E-05	0.00023053	FABP5/ACLY/MAPK1/ARG1/TTR/PSEN1/HEXA/HEXB/FUCA1/GGH/FPR1/PRCP/DPP7	13
CC	GO:0031256	leading edge membrane	3.785779336	5.437734191	2.22E-05	0.000239972	EGFR/GABRA5/AKT2/DPP4/ADORA1/ADORA2A/MAPT/SRC/PDE4A/ITGAV/ADAM17/OPRD1/ITGB1/FAP	14
CC	GO:0005641	nuclear envelope lumen	19.36155718	8.43530459	3.42E-05	0.000359942	PTGES/ALOX5/BCHE/APP	4
CC	GO:0070820	tertiary granule	3.836893953	5.29839851	3.78E-05	0.000388395	PTPN6/MMP9/PTAFR/MMP8/CDA/GGH/CTSD/ADAM10/FPR2/FPR1/PLAU/PRCP/LTA4H	13
CC	GO:0031252	cell leading edge	2.49973156	4.54465373	8.48E-05	0.000851493	PIK3CA/EGFR/GABRA5/AKT2/AKT1/DPP4/ADORA1/ADORA2A/CDK6/MYLK/MAPT/SRC/PDE4A/ITGAV/ADAM17/OPRD1/ITGB1/HDAC6/TLR4/DPP9/SLC9A1/FAP	22
CC	GO:0099572	postsynaptic specialization	2.696991102	4.590850937	9.61E-05	0.00094367	FABP5/SIGMAR1/GRIK2/EGLN1/AURKA/GABRA5/PDE4B/PTPRS/NTRK2/FYN/SRC/NOS1/OPRD1/ATP1A1/CHRM1/ADRA2A/ADAM10/GRIA2/GRIN1	19
CC	GO:0098687	chromosomal region	2.528561572	4.496875867	0.000104296	0.001001244	TERT/CDK2/AURKA/SIRT2/PARP1/CHEK1/AURKB/CDK1/TOP2A/APEX1/PRKDC/PSEN1/PSEN2/PPP1CC/PCNA/HSF1/PPP2CA/KDM4A/KDM4C/BLM/KDM1A	21
CC	GO:0035578	azurophil granule lumen	4.787198203	5.259159883	0.000110417	0.001036961	FABP5/ACLY/MAPK1/ARG1/TTR/HEXB/FUCA1/GGH/DPP7	9
CC	GO:1904724	tertiary granule lumen	6.160495466	5.566163945	0.000131745	0.001210932	PTPN6/MMP9/MMP8/CDA/GGH/CTSD/LTA4H	7
CC	GO:0000781	chromosome, telomeric region	3.357495464	4.523270789	0.00026335	0.002370154	TERT/CDK2/SIRT2/PARP1/CHEK1/CDK1/APEX1/PRKDC/PPP1CC/PCNA/BLM/KDM1A	12
CC	GO:0005788	endoplasmic reticulum lumen	2.628965431	4.219002087	0.000298755	0.002581246	CES2/PTGS2/MAPK3/BCHE/CES1/MAPK1/F10/F9/FLT3/F2/APP/IL6/ADAM17/ERAP2/ERAP1/STS/ADAM10	17
CC	GO:00	postsynaptic density	2.62896543	4.21900208	0.00029875	0.00258124	FABP5/SIGMAR1/GRIK2/EGLN1/AURKA/PDE4B/PTPRS/NTRK2/FYN/NOS1/	17

	14069		1	7	5	6	OPRD1/ATP1A1/CHRM1/ADRA2A/ADAM10/GRIA2/GRIN1	
MF	GO:0004879	nuclear receptor activity	20.79955081	21.53800499	2.51E-26	1.84E-23	RORC/AR/PPARG/PPARA/PPARD/ESR2/NR1H3/NR3C1/NR1H4/PGR/NR3C2/ESR1/RORA/NR1I3/NR4A2/ESRRA/AHR/VDR/NR1H2/STAT3/NR1I2/THRB/NR4A1/RORB	24
MF	GO:0098531	ligand-activated transcription factor activity	20.40710646	21.31370468	4.50E-26	1.84E-23	RORC/AR/PPARG/PPARA/PPARD/ESR2/NR1H3/NR3C1/NR1H4/PGR/NR3C2/ESR1/RORA/NR1I3/NR4A2/ESRRA/AHR/VDR/NR1H2/STAT3/NR1I2/THRB/NR4A1/RORB	24
MF	GO:0016705	oxidoreductase activity, acting on paired donors, with incorporation or reduction of molecular oxygen	7.804673164	13.78099307	4.65E-19	1.07E-16	NOS2/SCD/PTGS2/CYP19A1/CYP51A1/PTGS1/CYP17A1/CYP2C19/PHF8/KDM5C/EGLN1/CYP2C9/TYR/CYP1B1/AKR1C2/AKR1C1/AKR1C4/AKR1C3/CYP1A2/CYP1A1/SQLE/NOS1/NOS3/CYP2D6/KDM4A/KDM3A/KDM6B/FTO/KDM4C/KDM1A/CYP3A4	31
MF	GO:0004674	protein serine/threonine kinase activity	4.85485222	12.1401566	5.25E-19	1.07E-16	PRKCH/MAPK3/TOP1/MTOR/PIK3CA/MAPK8/CDK2/AURKA/CDK9/MAPK1/EGFR/MAPK14/MAPK9/SYK/MKNK2/MAP2K1/AKT2/AKT1/AKT3/TGFBR1/PIK3CG/MAPK10/CHEK1/PIM1/PIM2/MAP3K14/CDK6/GSK3B/AURKB/CDK1/MYLK/PKN1/PRKDC/IKBKB/CCND3/BRD4/EIF2AK3/PRKCA/PIM3/LRRK2/RPS6KA3/CLK4/CHUK/CSNK2B/CLK1/GRK5	46
MF	GO:0004953	icosanoid receptor activity	36.05255474	20.45875515	5.21E-18	8.51E-16	PPARG/LTB4R/PTGER2/PTGER4/PTGIR/PTGFR/PTGER3/PTGDR/PTGER1/PTGDR2/HPGD/CYSLTR2	12
MF	GO:0004955	prostaglandin receptor activity	45.06569343	20.99693237	2.60E-17	3.54E-15	PPARG/PTGER2/PTGER4/PTGIR/PTGFR/PTGER3/PTGDR/PTGER1/PTGDR2/HPGD	10
MF	GO:0004954	prostanoid receptor activity	40.96881221	19.97489733	2.80E-16	3.27E-14	PPARG/PTGER2/PTGER4/PTGIR/PTGFR/PTGER3/PTGDR/PTGER1/PTGDR2/HPGD	10
MF	GO:0004089	carbonate dehydratase activity	33.04817518	18.7052206	7.09E-16	7.24E-14	CA2/CA1/CA7/CA4/CA12/CA9/CA13/CA14/CA5A/CA3/CA6	11
MF	GO:00	steroid binding	9.26584350	12.9174002	1.62E-15	1.47E-13	RORC/HSD11B1/CD81/AR/HSD11B2/SERPINA6/SHBG/ESR2/NPC1L1/NR1H	22

	05496		9	8			3/NR3C1/NR1H4/PGR/NR3C2/ESR1/RORA/SMO/CETP/ESRRA/VDR/ATP1A1/CYP3A4	
MF	GO:0004713	protein tyrosine kinase activity	7.837511901	12.14505663	4.50E-15	3.68E-13	ALK/EGFR/MET/SYK/NTRK1/FLT1/MAP2K1/BTK/CSF1R/FLT3/KDR/LCK/NTRK2/FYN/SRC/PTK2/JAK1/JAK2/RET/CLK4/NTRK3/PDGFRA/CLK1/PDGFRB	24
MF	GO:0106310	protein serine kinase activity	4.593472884	10.41606381	1.04E-14	7.71E-13	PRKCH/MAPK3/MTOR/PIK3CA/MAPK8/CDK2/AURKA/CDK9/MAPK1/MAPK14/MAPK9/PIK3CB/MKNK2/MAP2K1/AKT2/AKT1/AKT3/PIK3CG/MAPK10/CHEK1/PIM1/PIM2/MAP3K14/CDK6/GSK3B/AURKB/CDK1/PKN1/PRKDC/IKBKB/EIF2AK3/PRKCA/PIM3/LRRK2/RPS6KA3/CLK4/CLK1	37
MF	GO:0016709	oxidoreductase activity, acting on paired donors, with incorporation or reduction of molecular oxygen, NAD(P)H as one donor, and incorporation of one atom of oxygen	16.60315021	14.50400762	3.36E-14	2.28E-12	NOS2/CYP51A1/CYP2C19/CYP2C9/AKR1C2/AKR1C1/AKR1C4/AKR1C3/CYP1A1/SQLE/NOS1/NOS3/CYP2D6/CYP3A4	14
MF	GO:0061629	RNA polymerase II-specific DNA-binding transcription factor binding	4.335766423	9.393763231	1.53E-12	9.31E-11	AR/PPARG/PPARA/NR1H4/ESR1/MAPK14/HDAC1/PARP1/NR4A2/FLT3/GSK3B/PKN1/PRKDC/AHR/SRC/VDR/NR1H2/CREBBP/STAT3/JUN/PCNA/NR1I2/HSF1/KDM3A/KDM4C/FBP1/TRIM24/KDM1A/NR4A1/CSNK2B/HDAC5/NFE2L2/KEAP1	33
MF	GO:0004497	monooxygenase activity	8.154744526	11.07544661	1.60E-12	9.31E-11	NOS2/CYP19A1/CYP51A1/CYP17A1/CYP2C19/CYP2C9/TYR/CYP1B1/AKR1C2/AKR1C1/AKR1C4/AKR1C3/CYP1A2/CYP1A1/SQLE/NOS1/NOS3/CYP2D6/CYP3A4	19
MF	GO:0032052	bile acid binding	36.05255474	16.70224758	2.38E-12	1.27E-10	PLA2G1B/FABP1/NR1H4/AKR1C2/AKR1C1/AKR1C4/AKR1C3/VDR	8

MF	GO:0140297	DNA-binding transcription factor binding	3.676908041	8.932291816	2.48E-12	1.27E-10	AR/PPARG/PPARA/PPARD/MAPK3/NR1H4/ESR1/MAPK14/SIRT2/HDAC1/PARP1/NR4A2/CCNT1/FLT3/GSK3B/PKN1/PRKDC/AHR/SRC/VDR/NR1H2/CREBBP/STAT3/JUN/HDAC8/PCNA/NR1I2/HSF1/KDM3A/KDM4C/FBP1/TRIM24/KDM1A/KLF5/NR4A1/CSNK2B/HDAC5/NFE2L2/KEAP1	39
MF	GO:0019902	phosphatase binding	5.722627737	9.830593946	5.51E-12	2.65E-10	PTPN1/PPARA/MAPK3/SLC6A3/MAPK8/MAPK1/EGFR/MAPK14/MET/GRB2/SYK/CSF1R/MAPT/LCK/PTK2/JAK1/EIF2AK3/PTPA/STAT3/PPP1CC/PIK3R1/HSP90AA1/SLC9A1/SPHK1	24
MF	GO:0034056	estrogen response element binding	32.77504977	15.87996545	8.55E-12	3.88E-10	AR/ESR2/NR3C1/PGR/NR3C2/ESR1/ESRRA/TRIM24	8
MF	GO:0030594	neurotransmitter receptor activity	7.817518248	10.19364009	4.88E-11	1.93E-09	CHRM2/GRIK2/CHRNA7/GABRA5/DRD2/GLRA1/GLRA2/DRD1/DRD3/CHRM3/CHRM1/HTR2B/HTR2C/HTR1B/DRD4/GRIA2/GRIN1	17
MF	GO:0016836	hydro-lyase activity	10.34294603	11.01039902	4.91E-11	1.93E-09	CA2/CA1/CA7/CA4/CA12/CA9/CYP1B1/CA13/CYP1A2/CYP1A1/CA14/CA5A/CA3/CA6	14
MF	GO:0019903	protein phosphatase binding	6.347280765	9.635767624	4.97E-11	1.93E-09	PTPN1/SLC6A3/MAPK8/EGFR/MAPK14/MET/GRB2/CSF1R/MAPT/LCK/PTK2/JAK1/EIF2AK3/PTPA/STAT3/PPP1CC/PIK3R1/HSP90AA1/SLC9A1/SPHK1	20
MF	GO:0016835	carbon-oxygen lyase activity	8.666479506	10.22109995	1.48E-10	5.48E-09	POLB/CA2/CA1/CA7/CA4/CA12/CA9/CYP1B1/CA13/CYP1A2/CYP1A1/CA14/CA5A/CA3/CA6	15
MF	GO:0042562	hormone binding	7.837511901	9.904014165	1.75E-10	6.21E-09	AR/SHBG/NR3C1/EGFR/AVPR1A/HCRTR2/HCRTR1/TTR/ATP1A1/THRB/ADRA2A/ADRA2B/DRD4/PIK3R1/SLC40A1/PDE3A	16
MF	GO:0097110	scaffold protein binding	9.013138686	10.11840056	3.52E-10	1.20E-08	PDE4D/GRIK2/SYK/MAP2K1/KCNH2/IKBKB/FYN/SRC/NOS1/NOS3/HSP90AA1/CHUK/ATG4B/CACNA1H	14
MF	GO:0020037	heme binding	6.029916727	9.063879545	3.74E-10	1.22E-08	NOS2/PTGS2/CYP19A1/CYP51A1/PTGS1/CYP17A1/CYP2C19/CYP2C9/CYP1B1/NOX4/CYP1A2/CYP1A1/SRC/NOS1/NOS3/JAK2/IDO1/CYP2D6/CYP3A4	19
MF	GO:0031406	carboxylic acid binding	5.058394161	8.604592953	4.82E-10	1.51E-08	PLA2G1B/NOS2/FABP1/PPARG/FABP4/PPARD/FABP5/NR1H4/FABP2/EGLN1/TYMS/AKR1C2/AKR1C1/AKR1C4/AKR1C3/VDR/NOS1/NOS3/GLRA1/GLRA2/DHFR/GRIN1	22

MF	GO:0004714	transmembrane receptor protein tyrosine kinase activity	9.764233577	10.24311637	5.26E-10	1.59E-08	ALK/EGFR/MET/NTRK1/FLT1/CSF1R/FLT3/KDR/NTRK2/RET/NTRK3/PDGFRA/PDGFRB	13
MF	GO:0001221	transcription coregulator binding	6.720322705	9.228934506	5.73E-10	1.67E-08	AR/PPARG/TERT/PPARA/PPARD/NR1H4/PGR/ESR1/RORA/CDK9/HDAC1/AHR/CREBBP/HDAC6/THRB/HDAC5/NFE2L2	17
MF	GO:0032451	demethylase activity	12.39306569	10.8662083	7.29E-10	2.05E-08	CYP51A1/PHF8/KDM5C/CYP1A2/CYP1A1/KDM4A/KDM3A/KDM6B/FTO/KDM4C/KDM1A	11
MF	GO:0004175	endopeptidase activity	3.600609527	7.798770096	8.08E-10	2.20E-08	PREP/MME/MMP9/CFD/F10/F9/REN/DPP4/MMP2/MMP13/F2/MMP3/PSEN1/ADAM17/PSEN2/CASP3/ERAP2/ERAP1/MMP12/MMP8/MMP1/MMP14/CTSL/CTSB/CTSD/ADAM10/PLAU/CAPN1/ATG4B/PRSS1/FAP	31
MF	GO:0046906	tetrapyrrole binding	5.633211679	8.6403487	1.21E-09	3.20E-08	NOS2/PTGS2/CYP19A1/CYP51A1/PTGS1/CYP17A1/CYP2C19/CYP2C9/CYP1B1/NOX4/CYP1A2/CYP1A1/SRC/NOS1/NOS3/JAK2/IDO1/CYP2D6/CYP3A4	19
MF	GO:0005506	iron ion binding	5.596393302	8.600071322	1.36E-09	3.39E-08	SCD/CYP19A1/CYP51A1/CYP17A1/CYP2C19/ALOX5/PHF8/EGLN1/CYP2C9/ALOX15/XDH/ALOX12/CYP1B1/CYP1A2/CYP1A1/CYP2D6/KDM3A/FTO/CYP3A4	19
MF	GO:0140993	histone modifying activity	4.789590606	8.259541087	1.37E-09	3.39E-08	PHF8/KDM5C/CDK2/AURKA/SIRT2/HDAC1/CHEK1/CDK1/PKN1/PRKDC/JAK2/CREBBP/PRKCA/HDAC6/HDAC8/KDM4A/KDM3A/KDM6B/KDM4C/KDM1A/HDAC5/WDR5	22
MF	GO:0043177	organic acid binding	4.766563728	8.229383357	1.50E-09	3.61E-08	PLA2G1B/NOS2/FABP1/PPARG/FABP4/PPARD/FABP5/NR1H4/FABP2/EGLN1/TYMS/AKR1C2/AKR1C1/AKR1C4/AKR1C3/VDR/NOS1/NOS3/GLRA1/GLRA2/DHFR/GRIN1	22
MF	GO:0003707	nuclear steroid receptor activity	16.22364964	11.47413745	1.78E-09	4.11E-08	PPARA/PPARD/ESR2/NR3C1/PGR/NR3C2/ESR1/ESRRA/PDE3A	9
MF	GO:0004032	alditol:NADP+ 1-oxidoreductase activity	26.28832117	13.20046785	1.81E-09	4.11E-08	AKR1B10/AKR1B1/AKR1C2/AKR1C1/AKR1C4/AKR1A1/AKR1C3	7
MF	GO:00	transmembrane receptor	7.98632541	9.37407631	1.89E-09	4.17E-08	ALK/EGFR/MET/NTRK1/FLT1/TGFBR1/CSF1R/FLT3/KDR/NTRK2/RET/NTR	14

	19199	protein kinase activity	8	8			K3/PDGFRA/PDGFRB	
MF	GO:0001223	transcription coactivator binding	11.26642336	10.27069749	2.23E-09	4.80E-08	AR/TERT/PPARA/PPARD/PGR/ESR1/RORA/CDK9/AHR/CREBBP/THRB	11
MF	GO:0017171	serine hydrolase activity	4.853228523	8.148511705	2.57E-09	5.38E-08	PREP/MMP9/CFD/F10/F9/DPP4/ACHE/MMP2/MMP13/F2/MMP3/MMP12/MMP8/MMP1/MMP14/DPP9/PLAU/PRSS1/PRCP/DPP7/FAP	21
MF	GO:0008528	G protein-coupled peptide receptor activity	6.080291971	8.619739798	2.79E-09	5.65E-08	LTB4R/AGTR1/AGTR2/AVPR1A/HCRTR2/HCRTR1/AVPR2/NTSR1/OPRM1/OPRD1/OPRK1/S1PR2/FPR2/FPR1/NTSR2/CYSLTR2/TACR2	17
MF	GO:0042578	phosphoric ester hydrolase activity	3.600289558	7.537216179	2.90E-09	5.65E-08	PTPN1/CDC25B/PTPRF/ACP1/PDE4D/PTPN6/PTPN11/CDC25A/CCR1/PDE4B/PDE8B/PDE5A/PTPRS/APEX1/LCK/PTK2/PDE4A/ENPP2/PTPA/ATP1A1/PPP1CC/CHRM3/CHRM1/PFKFB3/PPM1B/PPP2CA/FBP1/PDE3A/NT5E	29
MF	GO:0016229	steroid dehydrogenase activity	12.87591241	10.59374949	2.90E-09	5.65E-08	HSD11B1/HSD11B2/SRD5A2/AKR1C2/AKR1C1/AKR1C4/AKR1C3/SRD5A1/HSD17B3/HSD17B10	10
MF	GO:0001653	peptide receptor activity	5.803915063	8.343981502	5.75E-09	1.09E-07	LTB4R/AGTR1/AGTR2/AVPR1A/HCRTR2/HCRTR1/AVPR2/NTSR1/OPRM1/OPRD1/OPRK1/S1PR2/FPR2/FPR1/NTSR2/CYSLTR2/TACR2	17
MF	GO:0016922	nuclear receptor binding	5.633211679	8.169394717	9.11E-09	1.69E-07	PPARG/NR1H4/ESR1/PARP1/NR4A2/FLT3/PKN1/SRC/VDR/NR1H2/PCNA/NR1I2/KDM3A/KDM4C/TRIM24/KDM1A/NR4A1	17
MF	GO:0008236	serine-type peptidase activity	4.718920778	7.782559387	1.00E-08	1.82E-07	PREP/MMP9/CFD/F10/F9/DPP4/MMP2/MMP13/F2/MMP3/MMP12/MMP8/MMP1/MMP14/DPP9/PLAU/PRSS1/PRCP/DPP7/FAP	20
MF	GO:0033218	amide binding	3.305552085	7.102697356	1.11E-08	1.98E-07	PTGES/PPARG/SRD5A2/BCHE/CHRNA7/MME/AVPR1A/TYMS/HCRTR2/HCRTR1/ACHE/AVPR2/PLA2G4A/PTGDR2/SRD5A1/OPRM1/OPRD1/OPRK1/LNPEP/ERAP2/ERAP1/DHFR/NQO2/TLR4/HSP90AB1/PIK3R1/FPR2/GRIA2/GRIN1/SLC40A1	30
MF	GO:0051213	dioxygenase activity	6.784082882	8.423779849	1.70E-08	2.95E-07	PTGS2/PTGS1/ALOX5/PHF8/KDM5C/EGLN1/ALOX15/ALOX12/IDO1/KDM4A/KDM3A/KDM6B/FTO/KDM4C	14
MF	GO:0016616	oxidoreductase activity, acting on the CH-OH	5.768408759	8.05817864	1.77E-08	3.01E-07	AKR1B10/HSD11B1/HSD11B2/G6PD/HMGCR/SRD5A2/AKR1B1/AKR1C2/AKR1C1/AKR1C4/AKR1A1/AKR1C3/CBR1/HSD17B3/HPGD/HSD17B10	16

		group of donors, NAD or NADP as acceptor						
MF	GO:0008106	alcohol dehydrogenase (NADP+) activity	15.6750238	10.60841541	2.01E-08	3.29E-07	AKR1B10/AKR1B1/AKR1C2/AKR1C1/AKR1C4/AKR1A1/AKR1C3/CBR1	8
MF	GO:0035173	histone kinase activity	15.6750238	10.60841541	2.01E-08	3.29E-07	CDK2/AURKA/CHEK1/CDK1/PKN1/PRKDC/JAK2/PRKCA	8

Table S2. The top 50 significantly enriched KEGG pathways for the shared targets of Pg and reproductive system injury.

category	subcategory	ID	Description	pvalue	p.adjust	qvalue	geneID	Count
Environmental Information Processing	Signaling molecules and interaction	hsa04080	Neuroactive ligand-receptor interaction	1.61E-18	5.02E-16	2.18E-16	LTB4R/PTGER2/ADORA3/NR3C1/PTGER4/CHRM2/GRIK2/PTGIR/CHRNA7/PTGFR/S1PR1/AGTR1/GABRA5/AGTR2/P2RX3/AVPR1A/HCRTR2/HCRTR1/S1PR3/ADORA1/ADORA2A/AVPR2/F2/PTGER3/PTGDR/PTGER1/DRD2/CNR1/NTSR1/OPRM1/OPRD1/OPRK1/GLRA1/GLRA2/PTAFR/DRD1/DRD3/THRB/CHRM3/CHRM1/S1PR2/CNR2/HTR2B/ADRA2A/ADRA2B/HTR2C/HTR1B/P2RY4/P2RY2/DRD4/FPR2/GRIA2/C5AR1/FPR1/NTSR2/GRIN1/CYSLTR2/PRSS1/TACR2	59
Environmental Information Processing	Signal transduction	hsa04151	PI3K-Akt signaling pathway	4.61E-15	1.59E-13	6.91E-14	MAPK3/CHRM2/MDM2/MTOR/PIK3CA/CDK2/MAPK1/EGFR/MET/GRB2/PIK3CB/SYK/NTRK1/FLT1/MAP2K1/AKT2/AKT1/AKT3/PIK3CD/PIK3CG/CSF1R/FLT3/KDR/CDK6/GSK3B/PKN1/IKBKB/NTRK2/PTK2/CCNE2/CCND3/ITGAV/IL6/NOS3/JAK1/JAK2/BCL2L1/PRKCA/ITGA2B/ITGB1/IL2/CHRM1/RET/PPP2CA/NFKB1/TLR4/HSP90AB1/PIK3R1/HSP90AA1/NR4A1/PDGFRA/CHUK/PDGFRB	53
Human Diseases	Cardiovascular disease	hsa05417	Lipid and atherosclerosis	8.93E-16	5.56E-14	2.41E-14	PPARG/MAPK3/PIK3CA/MAPK8/MAPK1/MAPK14/MAPK9/PIK3CB/MMP9/CYP2C9/AKT2/AKT1/AKT3/PIK3CD/MAPK10/GSK3B/CALM1/IKBKB/CYP1A1/SRC/PTK2/TNF/MMP3/IL6/NOS3/JAK2/EIF2AK3/BCL2L1/PRKCA/CASP3/STAT3/JUN/MMP1/NFKB1/TLR4/HSP90AB1/PIK3R1/HSP90AA1/CHUK/NFE2L2/NOX1	41
Human Diseases	Cancer: overview	hsa05208	Chemical carcinogenesis - reactive oxygen species	5.43E-15	1.69E-13	7.32E-14	PTPN1/ACP1/PTPN11/MAPK3/PIK3CA/MAPK8/MAPK1/EGFR/MAPK14/MET/GRB2/MAPK9/PIK3CB/MAP2K1/AKT2/AKT1/AKT3/PIK3CD/MAPK10/MAP3K14/CYP1B1/AKR1C2/AKR1C1/AKR1C4/AKR1A1/NOX4/AHR/AKR1C3/IKBKB/CYP1A2/CBR1/CYP1A1/SRC/P	41

							TK2/JUN/NFKB1/PIK3R1/CHUK/NFE2L2/NOX1/KEAP1	
Human Diseases	Cancer: overview	hsa05207	Chemical carcinogenesis - receptor activation	2.27E-14	5.42E-13	2.35E-13	AR/PPARA/CDC25A/ESR2/MAPK3/PGR/ESR1/NR1I3/CHRNA7/UGT2B7/MTOR/PIK3CA/MAPK1/EGFR/GRB2/PIK3CB/MAP2K1/AKT2/AKT1/AKT3/PIK3CD/CYP1B1/AHR/CYP1A2/CYP1A1/SRC/VDR/CCND3/JAK2/PRKCA/STAT3/JUN/RPS6KA3/NFKB1/KLF5/HSP90AB1/PIK3R1/HSP90AA1/CYP3A4	39
Environmental Information Processing	Signal transduction	hsa04024	cAMP signaling pathway	1.23E-13	1.88E-12	8.14E-13	PDE4D/PPARA/PTGER2/MAPK3/CHRM2/PIK3CA/MAPK8/MAPK1/MAPK9/PIK3CB/MAP2K1/AKT2/AKT1/AKT3/PIK3CD/MAPK10/PDE4B/ADORA1/ADORA2A/CALM1/PTGER3/DRD2/PDE4A/CREBBP/GLI1/JUN/ATP1A1/PPP1CC/DRD1/CHRM1/HTR1B/GPR119/NFKB1/PIK3R1/GRIA2/GRIN1/SLC9A1/PDE3A/CFTR	39
Human Diseases	Infectious disease: viral	hsa05161	Hepatitis B	2.52E-15	9.79E-14	4.24E-14	MAPK3/PIK3CA/MAPK8/CDK2/MAPK1/MAPK14/GRB2/MAPK9/PIK3CB/MMP9/MAP2K1/AKT2/AKT1/AKT3/TGFBR1/PIK3CD/MAPK10/IKBKB/SRC/TNF/CCNE2/IL6/JAK1/JAK2/CREBBP/PRKCA/CASP3/SLC10A1/STAT3/JUN/PCNA/NFKB1/TLR4/PIK3R1/CHUK	35
Human Diseases	Cardiovascular disease	hsa05418	Fluid shear stress and atherosclerosis	5.36E-14	1.01E-12	4.36E-13	PIK3CA/MAPK8/MAPK14/MAPK9/PIK3CB/MMP9/AKT2/AKT1/AKT3/PIK3CD/MAPK10/KDR/MMP2/CALM1/IKBKB/SRC/PTK2/TNF/ITGAV/NOS3/ITGA2B/JUN/CTSL/NFKB1/HSP90AB1/PIK3R1/HSP90AA1/CHUK/NFE2L2/NOX1/KEAP1	31
Human Diseases	Cancer: specific types	hsa05215	Prostate cancer	7.07E-18	1.10E-15	4.76E-16	AR/MAPK3/SRD5A2/MDM2/MTOR/PIK3CA/CDK2/MAPK1/EGFR/GRB2/PIK3CB/MMP9/MAP2K1/AKT2/AKT1/AKT3/PIK3CD/GSK3B/IKBKB/CCNE2/MMP3/CREBBP/NFKB1/HSP90AB1/PIK3R1/HSP90AA1/PDGFRA/CHUK/PLAU/PDGFRB	30
Human Diseases	Endocrine and metabolic disease	hsa04933	AGE-RAGE signaling pathway in diabetic complications	1.77E-16	1.84E-14	7.96E-15	MAPK3/PIK3CA/MAPK8/MAPK1/MAPK14/MAPK9/PIK3CB/AGTR1/AKT2/AKT1/AKT3/TGFBR1/PIK3CD/MAPK10/PIM1/MMP2/NOX4/TNF/IL6/NOS3/JAK2/PRKCA/CASP3/STAT3/JUN/SERPINE1/NFK	29

							B1/PIK3R1/NOX1	
Human Diseases	Endocrine and metabolic disease	hsa04931	Insulin resistance	1.68E-15	8.70E-14	3.77E-14	PTPN1/PTPRF/PTPN11/PPARA/NR1H3/MTOR/PIK3CA/MAPK8/MAPK9/PIK3CB/AKT2/AKT1/AKT3/PIK3CD/MAPK10/GSK3B/IKBKB/TNF/NR1H2/IL6/NOS3/PTPA/STAT3/PPP1CC/RPS6KA3/PYGM/OGT/NFKB1/PIK3R1	29
Environmental Information Processing	Signal transduction	hsa04071	Sphingolipid signaling pathway	4.19E-14	8.68E-13	3.76E-13	ADORA3/MAPK3/PIK3CA/MAPK8/MAPK1/MAPK14/MAPK9/PIK3CB/S1PR1/MAP2K1/AKT2/AKT1/AKT3/PIK3CD/MAPK10/S1PR3/ADORA1/ABCC1/FYN/TNF/NOS3/OPRD1/PRKCA/S1PR2/PPP2CA/NFKB1/CTSD/PIK3R1/SPHK1	29
Human Diseases	Cancer: overview	hsa05235	PD-L1 expression and PD-1 checkpoint pathway in cancer	5.92E-16	4.60E-14	1.99E-14	PTPN6/PTPN11/MAPK3/TLR9/MTOR/PIK3CA/MAPK1/ALK/EGFR/MAPK14/PIK3CB/MAP2K1/AKT2/AKT1/AKT3/PIK3CD/LCK/IKBKB/JAK1/JAK2/STAT3/JUN/NFKB1/TLR4/PIK3R1/CHUK/CSNK2B	27
Human Diseases	Drug resistance: antineoplastic	hsa01522	Endocrine resistance	8.34E-15	2.36E-13	1.02E-13	ESR2/MAPK3/ESR1/MDM2/MTOR/PIK3CA/MAPK8/MAPK1/EGFR/MAPK14/GRB2/MAPK9/PIK3CB/MMP9/MAP2K1/ABCB11/AKT2/AKT1/AKT3/PIK3CD/MAPK10/MMP2/SRC/PTK2/JUN/CYP2D6/PIK3R1	27
Organismal Systems	Immune system	hsa04625	C-type lectin receptor signaling pathway	4.09E-14	8.68E-13	3.76E-13	PTPN11/PTGS2/MAPK3/MDM2/PIK3CA/MAPK8/MAPK1/MAPK14/MAPK9/PIK3CB/SYK/AKT2/AKT1/AKT3/PIK3CD/MAPK10/MAP3K14/CALM1/IKBKB/SRC/TNF/IL6/JUN/IL2/NFKB1/PIK3R1/CHUK	27
Human Diseases	Drug resistance: antineoplastic	hsa01521	EGFR tyrosine kinase inhibitor resistance	2.51E-15	9.79E-14	4.24E-14	MAPK3/MTOR/PIK3CA/MAPK1/EGFR/MET/GRB2/PIK3CB/MAP2K1/AKT2/AKT1/AKT3/PIK3CD/KDR/GSK3B/SRC/IL6/JAK1/JAK2/BCL2L1/PRKCA/STAT3/PIK3R1/PDGFRA/PDGFRB	25
Organismal Systems	Endocrine system	hsa04917	Prolactin signaling pathway	1.40E-14	3.63E-13	1.57E-13	ESR2/CYP17A1/MAPK3/ESR1/PIK3CA/MAPK8/MAPK1/MAPK14/GRB2/MAPK9/PIK3CB/MAP2K1/AKT2/AKT1/AKT3/PIK3CD/MAPK10/GSK3B/SRC/JAK2/STAT3/NFKB1/PIK3R1	23
Human Diseases	Cancer:	hsa052	Acute myeloid	5.50E-14	1.01E-12	4.36E-13	PPARD/MAPK3/MTOR/PIK3CA/MAPK1/GRB2/PIK3CB/MAP2K1/	22

	specific types	21	leukemia				AKT2/AKT1/AKT3/PIK3CD/PIM1/PIM2/CSF1R/FLT3/IKBKB/STAT3/BCL2A1/NFKB1/PIK3R1/CHUK	
Organismal Systems	Immune system	hsa04664	Fc epsilon RI signaling pathway	7.75E-14	1.34E-12	5.80E-13	MAPK3/ALOX5/PIK3CA/MAPK8/MAPK1/MAPK14/GRB2/MAPK9/PIK3CB/SYK/MAP2K1/AKT2/AKT1/AKT3/PIK3CD/BTK/MAPK10/PLA2G4A/FYN/TNF/PRKCA/PIK3R1	22
Organismal Systems	Sensory system	hsa04750	Inflammatory mediator regulation of TRP channels	2.21E-10	1.49E-09	6.48E-10	PRKCH/PTGER2/PTGER4/PIK3CA/MAPK8/MAPK14/MAPK9/PIK3CB/NTRK1/PIK3CD/MAPK10/ALOX12/CALM1/PLA2G4A/SRC/TRPM8/PRKCA/PPP1CC/HTR2B/HTR2C/P2RY2/PIK3R1	22
Organismal Systems	Endocrine system	hsa04919	Thyroid hormone signaling pathway	1.42E-08	6.50E-08	2.82E-08	MAPK3/ESR1/MDM2/MTOR/PIK3CA/MAPK1/PIK3CB/MAP2K1/AKT2/HDAC1/AKT1/AKT3/PIK3CD/GSK3B/SRC/ITGAV/CREBBP/PRKCA/ATP1A1/THRB/PIK3R1/SLC9A1	22
Organismal Systems	Endocrine system	hsa04935	Growth hormone synthesis, secretion and action	1.42E-08	6.50E-08	2.82E-08	MAPK3/MTOR/PIK3CA/MAPK8/MAPK1/MAPK14/GRB2/MAPK9/PIK3CB/MAP2K1/AKT2/AKT1/AKT3/PIK3CD/MAPK10/GSK3B/PTK2/JAK2/CREBBP/PRKCA/STAT3/PIK3R1	22
Human Diseases	Endocrine and metabolic disease	hsa04936	Alcoholic liver disease	3.07E-07	1.20E-06	5.18E-07	FABP1/SCD/PPARA/MAPK8/MAPK14/MAPK9/AKT2/AKT1/AKT3/MAPK10/MAP3K14/GSK3B/NOX4/IKBKB/TNF/IL6/CASP3/NFKB1/TLR4/C5AR1/CHUK/ACACA	22
Human Diseases	Endocrine and metabolic disease	hsa04932	Non-alcoholic fatty liver disease	1.40E-06	4.59E-06	1.99E-06	PPARG/PPARA/NR1H3/PIK3CA/MAPK8/MAPK14/MAPK9/PIK3CB/AKT2/AKT1/AKT3/PIK3CD/MAPK10/GSK3B/IKBKB/TNF/IL6/EIF2AK3/CASP3/JUN/NFKB1/PIK3R1	22
Human Diseases	Cancer: specific types	hsa05225	Hepatocellular carcinoma	5.37E-06	1.65E-05	7.16E-06	TERT/MAPK3/MTOR/PIK3CA/MAPK1/EGFR/MET/GRB2/PIK3CB/MAP2K1/AKT2/AKT1/AKT3/TGFBR1/PIK3CD/CDK6/GSK3B/BCL2L1/PRKCA/PIK3R1/NFE2L2/KEAP1	22
Environmental Information Processing	Signal transduction	hsa04012	ErbB signaling pathway	8.65E-11	6.41E-10	2.78E-10	MAPK3/MTOR/PIK3CA/MAPK8/MAPK1/EGFR/GRB2/MAPK9/PIK3CB/MAP2K1/AKT2/AKT1/AKT3/PIK3CD/MAPK10/GSK3B/SRC/PTK2/PRKCA/JUN/PIK3R1	21

Human Diseases	Cancer: specific types	hsa05222	Small cell lung cancer	4.18E-10	2.60E-09	1.13E-09	NOS2/PTGS2/PIK3CA/CDK2/PIK3CB/AKT2/AKT1/AKT3/PIK3CD/CDK6/IKBKB/PTK2/CCNE2/ITGAV/BCL2L1/ITGA2B/ITGB1/CASP3/NFKB1/PIK3R1/CHUK	21
Organismal Systems	Immune system	hsa04657	IL-17 signaling pathway	6.37E-10	3.74E-09	1.62E-09	PTGS2/MAPK3/MAPK8/MAPK1/MAPK14/MAPK9/MMP9/MAPK10/GSK3B/MMP13/IKBKB/TNF/MMP3/IL6/CASP3/JUN/MMP1/NFKB1/HSP90AB1/HSP90AA1/CHUK	21
Human Diseases	Cancer: overview	hsa05231	Choline metabolism in cancer	1.43E-09	7.40E-09	3.21E-09	MAPK3/MTOR/PIK3CA/MAPK8/MAPK1/EGFR/GRB2/MAPK9/PIK3CB/MAP2K1/AKT2/AKT1/AKT3/PIK3CD/MAPK10/PLA2G4A/PRKCA/JUN/PIK3R1/PDGFRA/PDGFRB	21
Organismal Systems	Nervous system	hsa04726	Serotonergic synapse	2.42E-08	1.06E-07	4.60E-08	PTGS2/PTGS1/MAPK3/SLC6A4/CYP2C19/ALOX5/MAPK1/MAOB/CYP2C9/MAP2K1/ALOX15/ALOX12/MAOA/PLA2G4A/APP/PRKCA/CASP3/HTR2B/HTR2C/CYP2D6/HTR1B	21
Cellular Processes	Transport and catabolism	hsa04140	Autophagy - animal	1.66E-05	4.82E-05	2.09E-05	MAPK3/MTOR/PIK3CA/MAPK8/MAPK1/MAPK9/PIK3CB/MAP2K1/AKT2/AKT1/AKT3/PIK3CD/MAPK10/EIF2AK3/BCL2L1/CTSL/CTSB/PPP2CA/CTSD/PIK3R1/ATG4B	21
Human Diseases	Infectious disease: bacterial	hsa05130	Pathogenic Escherichia coli infection	2.44E-04	5.98E-04	2.59E-04	PTPN6/PTPN11/MAPK3/MAPK8/MAPK1/MAPK14/MAPK9/MAPK10/F2/IKBKB/FYN/SRC/TNF/IL6/ITGB1/CASP3/JUN/TUBB1/NFKB1/TLR4/CHUK	21
Human Diseases	Cancer: overview	hsa05230	Central carbon metabolism in cancer	1.43E-11	1.27E-10	5.51E-11	G6PD/MAPK3/MTOR/PIK3CA/MAPK1/EGFR/MET/PIK3CB/NTRK1/MAP2K1/AKT2/AKT1/AKT3/PIK3CD/FLT3/RET/NTRK3/PIK3R1/PDGFRA/PDGFRB	20
Human Diseases	Cancer: specific types	hsa05220	Chronic myeloid leukemia	7.31E-11	5.55E-10	2.40E-10	PTPN11/MAPK3/MDM2/PIK3CA/MAPK1/GRB2/PIK3CB/MAP2K1/AKT2/HDAC1/AKT1/AKT3/TGFBR1/PIK3CD/CDK6/IKBKB/BCL2L1/NFKB1/PIK3R1/CHUK	20
Human Diseases	Cancer: specific types	hsa05210	Colorectal cancer	7.84E-10	4.52E-09	1.96E-09	MAPK3/MTOR/PIK3CA/MAPK8/MAPK1/EGFR/GRB2/MAPK9/PIK3CB/MAP2K1/AKT2/AKT1/AKT3/TGFBR1/PIK3CD/MAPK10/GSK3	20

							B/CASP3/JUN/PIK3R1	
Organismal Systems	Immune system	hsa04662	B cell receptor signaling pathway	1.83E-09	9.04E-09	3.92E-09	CD81/PTPN6/MAPK3/PIK3CA/MAPK1/GRB2/PIK3CB/SYK/MAP2K1/AKT2/AKT1/AKT3/PIK3CD/BTK/GSK3B/IKBKB/JUN/NFKB1/PIK3R1/CHUK	20
Organismal Systems	Nervous system	hsa04728	Dopaminergic synapse	1.22E-06	4.14E-06	1.79E-06	SLC6A3/MAPK8/MAPK14/MAPK9/MAOB/AKT2/AKT1/AKT3/MAPK10/GSK3B/MAOA/CALM1/DRD2/PRKCA/PPP1CC/DRD1/DRD3/PPP2CA/DRD4/GRIA2	20
Human Diseases	Cancer: specific types	hsa05226	Gastric cancer	9.16E-06	2.77E-05	1.20E-05	TERT/MAPK3/MTOR/PIK3CA/CDK2/MAPK1/EGFR/MET/GRB2/PIK3CB/MAP2K1/AKT2/AKT1/AKT3/TGFBR1/PIK3CD/ABCB1/GSK3B/CCNE2/PIK3R1	20
Environmental Information Processing	Signal transduction	hsa04022	cGMP-PKG signaling pathway	4.14E-05	1.14E-04	4.92E-05	ADORA3/MAPK3/MAPK1/AGTR1/MAP2K1/AKT2/AKT1/AKT3/PIK3CG/ADORA1/PDE5A/MYLK/CALM1/NOS3/OPRD1/ATP1A1/PPP1CC/ADRA2A/ADRA2B/PDE3A	20
Human Diseases	Neurodegenerative disease	hsa05020	Prion disease	2.19E-02	3.91E-02	1.69E-02	MAPK3/PIK3CA/MAPK8/MAPK1/MAPK14/MAPK9/PIK3CB/PIK3CD/MAPK10/GSK3B/FYN/TNF/IL6/EIF2AK3/CASP3/TUBB1/PIK3R1/GRIN1/CSNK2B/PSMB1	20
Environmental Information Processing	Signal transduction	hsa04370	VEGF signaling pathway	4.63E-12	4.24E-11	1.84E-11	PTGS2/MAPK3/PIK3CA/MAPK1/MAPK14/PIK3CB/MAP2K1/AKT2/AKT1/AKT3/PIK3CD/KDR/PLA2G4A/SRC/PTK2/NOS3/PRKCA/PIK3R1/SPHK1	19
Human Diseases	Cancer: specific types	hsa05214	Glioma	4.51E-10	2.74E-09	1.19E-09	MAPK3/MDM2/MTOR/PIK3CA/MAPK1/EGFR/GRB2/PIK3CB/MAP2K1/AKT2/AKT1/AKT3/PIK3CD/CDK6/CALM1/PRKCA/PIK3R1/PDGFRA/PDGFRB	19
Organismal Systems	Nervous system	hsa04725	Cholinergic synapse	6.69E-07	2.34E-06	1.01E-06	MAPK3/CHRM2/CHRNA7/PIK3CA/MAPK1/PIK3CB/MAP2K1/AKT2/AKT1/AKT3/PIK3CD/PIK3CG/ACHE/FYN/JAK2/PRKCA/CHRM3/CHRM1/PIK3R1	19
Environmental	Signal	hsa043	Apelin signaling	1.19E-05	3.51E-05	1.52E-05	NOS2/MAPK3/MTOR/MAPK1/AGTR1/MAP2K1/AKT2/AKT1/AKT	19

Information Processing	transduction	71	pathway				3/TGFBR1/PIK3CG/MYLK/CALM1/NOS1/NOS3/SERPINE1/HDAC5/SLC9A1/SPHK1	
Human Diseases	Cancer: specific types	hsa05224	Breast cancer	2.64E-05	7.55E-05	3.27E-05	ESR2/MAPK3/PGR/ESR1/MTOR/PIK3CA/MAPK1/EGFR/GRB2/PIK3CB/MAP2K1/AKT2/AKT1/AKT3/PIK3CD/CDK6/GSK3B/JUN/PIK3R1	19
Organismal Systems	Immune system	hsa04621	NOD-like receptor signaling pathway	6.83E-04	1.56E-03	6.77E-04	MAPK3/MAPK8/MAPK1/MAPK14/MAPK9/MAPK10/PKN1/IKBKB/TNF/IL6/JAK1/BCL2L1/JUN/CTSB/NFKB1/TLR4/HSP90AB1/HSP90AA1/CHUK	19
Human Diseases	Cancer: overview	hsa05202	Transcriptional misregulation in cancer	1.20E-03	2.68E-03	1.16E-03	PPARG/MDM2/CDK9/MET/MMP9/NTRK1/FLT1/HDAC1/CSF1R/CCNT1/FLT3/PTK2/MMP3/IL6/HPGD/BCL2L1/BCL2A1/NFKB1/PLAU	19
Human Diseases	Cancer: specific types	hsa05223	Non-small cell lung cancer	1.67E-09	8.37E-09	3.62E-09	MAPK3/PIK3CA/MAPK1/ALK/EGFR/MET/GRB2/PIK3CB/MAP2K1/AKT2/AKT1/AKT3/PIK3CD/CDK6/PRKCA/STAT3/RET/PIK3R1	18
Cellular Processes	Cell growth and death	hsa04110	Cell cycle	2.05E-04	5.10E-04	2.21E-04	CDC25B/CDC25A/MDM2/CDK2/HDAC1/CHEK1/CCNB3/CDK6/GSK3B/AURKB/CDK1/PRKDC/CCNE2/CCND3/CREBBP/HDAC8/PCNA/PPP2CA	18
Environmental Information Processing	Signal transduction	hsa04150	mTOR signaling pathway	2.05E-04	5.10E-04	2.21E-04	MAPK3/MTOR/PIK3CA/MAPK1/GRB2/PIK3CB/MAP2K1/AKT2/AKT1/AKT3/PIK3CD/GSK3B/IKBKB/TNF/PRKCA/RPS6KA3/PIK3R1/CHUK	18

Table S3. Effects of different BPA doses and exposure durations on sperm morphology in mice.

Group	Modeling Duration (days)	Total Sperm Counted	Normal Sperm Count	Abnormal Sperm Count	Head Defect	Midpiece Defect	Tail Defect	Excess Residual Cytoplasm	Spenn deformity index	Teratozoospermia Index
NC (7-day)	7	200	176	24	22	1	6	0	14.50%	1.208
BPA-50 (7-day)	7	200	169	31	20	8	8	0	18.00%	1.161
BPA-100 (7-day)	7	200	145	55	44	6	7	0	28.50%	1.036
BPA-200 (7-day)	7	200	123	77	56	8	15	3	39.50%	1.065
NC (14-day)	14	200	179	21	15	2	5	0	11.00%	1.048
BPA-50 (14-day)	14	200	177	23	21	3	7	1	15.50%	1.391
BPA-100 (14-day)	14	200	154	46	27	8	15	1	25.00%	1.109
BPA-200 (14-day)	14	200	125	75	52	4	22	2	39.00%	1.067

Note: 1. Group nomenclature: Groups are designated as follows: NC (Normal Control, vehicle only); BPA-50, BPA-100, BPA-200 (Bisphenol A at 50, 100, and 200 mg/kg body weight, respectively). The suffix in parentheses indicates the modeling duration (7 or 14 days). All treatments were administered daily via oral gavage (n=6).

2. Based on these results, we selected a daily oral gavage of 100 mg/kg BPA for 14 days to establish the reproductive injury model. This condition induced a substantial and reproducible increase in sperm deformity (25.0%) compared to the NC group (11.0%), establishing a well-defined injury model suitable for investigating protective interventions, while avoiding the excessive toxicity observed at the 200 mg/kg dose.

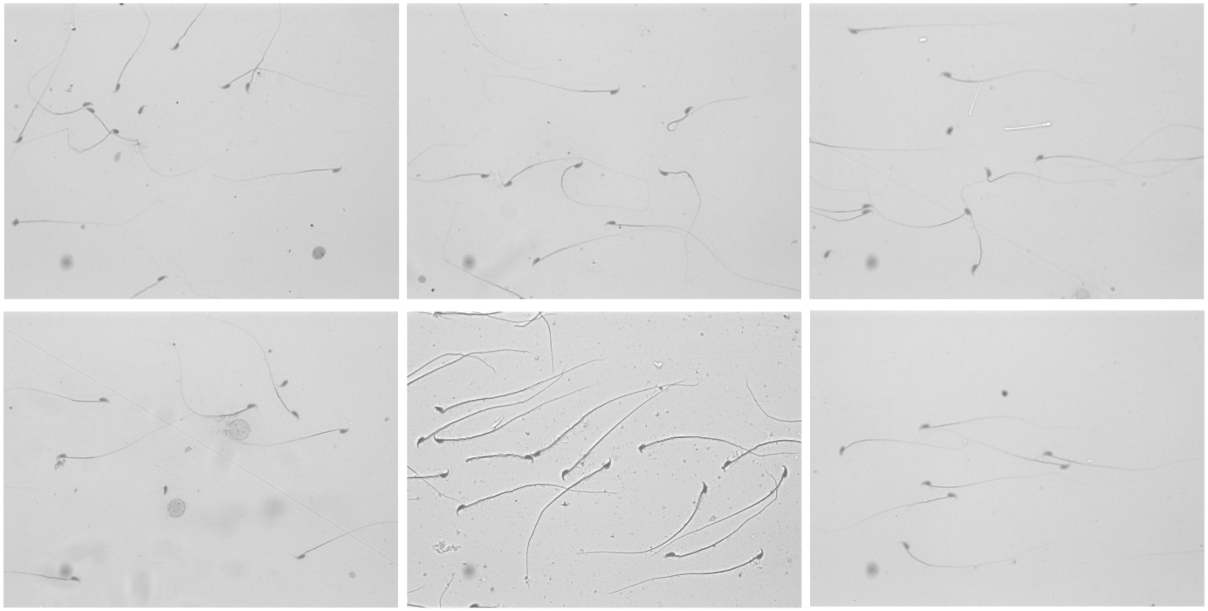

Figure S1. Assessment of sperm morphology in mice after 28-day administration of Pg (400 mg/kg).

Note: 1. Shown are representative sperm morphology micrographs from all six male ICR mice following a 28-day repeated dose toxicity study. Mice were orally administered with Pg at 400 mg/kg/day. Sperm samples were collected and examined for teratospermia. The images display normal sperm morphology across all subjects, with no evident increases in head or tail defects. Quantitative analysis confirmed no significant change in the sperm abnormality rate compared to controls, indicating a lack of reproductive toxicity at this high dose.

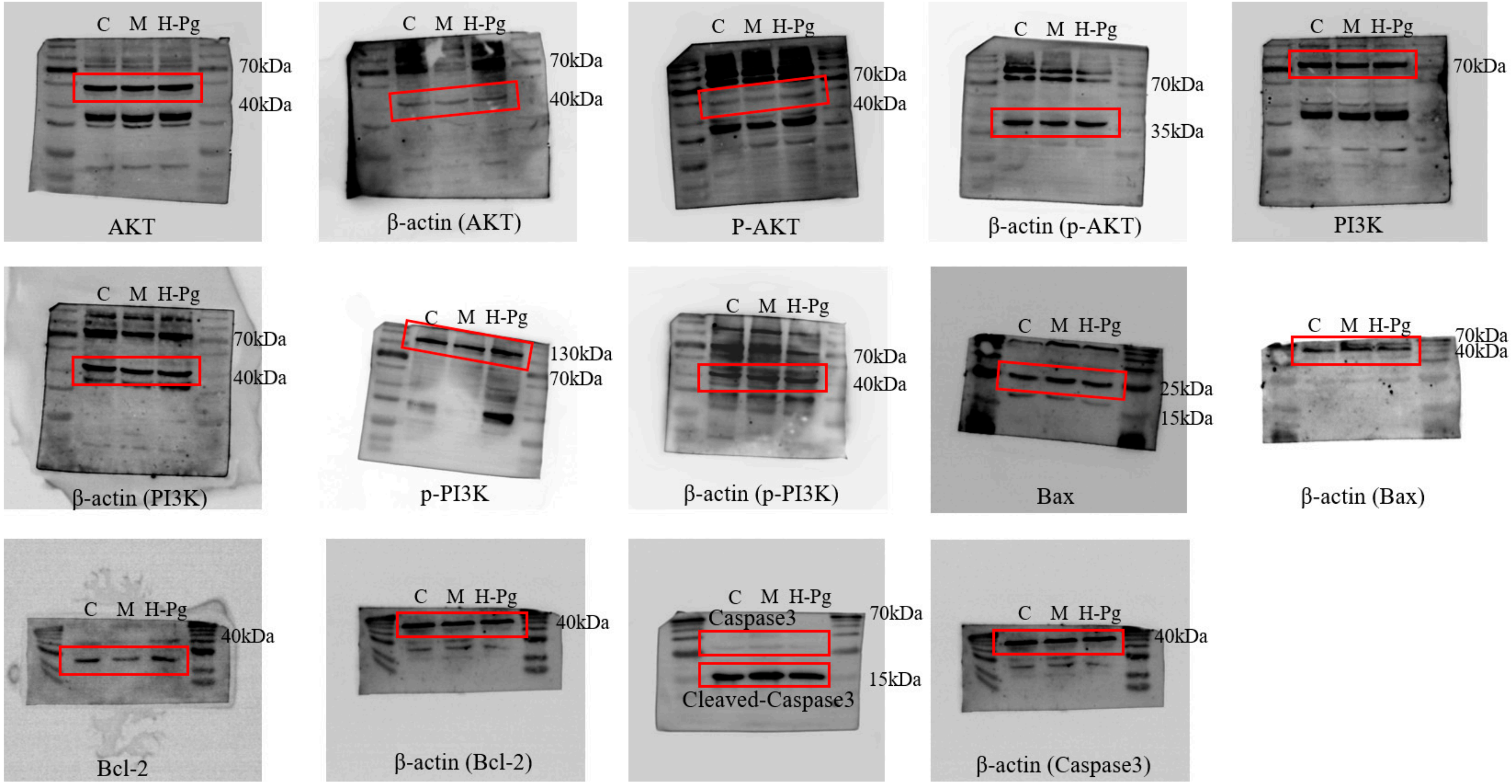

Figure S2. Original Western blot images.
